# Advances for Triangular and Sandwich-Shaped All-Metal Aromatics

**DOI:** 10.3390/molecules29040763

**Published:** 2024-02-07

**Authors:** Miaomiao Wang, Yanlan Wang

**Affiliations:** Department of Chemistry and Chemical Engineering, Shandong Provincial Key Laboratory of Chemical Energy Storage and Novel Cell Technology, Liaocheng University, Liaocheng 252059, China; wmm_0214@163.com

**Keywords:** all-metal aromaticity, coordination, catalysis

## Abstract

Much experimental work has been contributed to all-metal σ, π and *δ*-aromaticity among transition metals, semimetallics and other metals in the past two decades. Before our focused investigations on the properties of triangular and sandwich-shaped all-metal aromatics, A. I. Boldyrev presented general discussions on the concepts of all-metal σ-aromaticity and σ-antiaromaticity for metallo-clusters. Schleyer illustrated that Nucleus-Independent Chemical Shifts (NICS) were among the most authoritative criteria for aromaticity. Ugalde discussed the earlier developments of all-metal aromatic compounds with all possible shapes. Besides the theoretical predictions, many stable all-metal aromatic trinuclear clusters have been isolated as the metallic analogues of either the σ-aromatic molecule’s [H_3_]^+^ ion or the π-aromatic molecule’s [C_3_H_3_]^+^ ion. Different from Hoffman’s opinion on all-metal aromaticity, triangular all-metal aromatics were found to hold great potential in applications in coordination chemistry, catalysis, and material science. Triangular all-metal aromatics, which were theoretically proved to conform to the Hückel (4n + 2) rule and possess the smallest aromatic ring, could also play roles as stable ligands during the formation of all-metal sandwiches. The triangular and sandwich-shaped all-metal aromatics have not yet been specifically summarized despite their diversity of existence, puissant developments and various interesting applications. These findings are different from the public opinion that all-metal aromatics would be limited to further applications due to their overstated difficulties in synthesis and uncertain stabilities. Our review will specifically focus on the summarization of theoretical predictions, feasible syntheses and isolations, and multiple applications of triangular and sandwich shaped all-metal aromatics. The appropriateness and necessities of this review will emphasize and disseminate their importance and applications forcefully and in a timely manner.

## 1. Introduction

The definition for aromaticity was originally proposed in the 19th century when August Kekulé proposed the typical structure of benzene, C_6_H_6_ [1,2,3]. Since then, aromaticity has become a popular concept and was wildly used in organic chemistry to demonstrate molecules possessing conjugated (4n + 2) π electrons. After vigorous and long development, this concept has been extended to numerous unsaturated organic species including the smallest cyclopropenyl cation and much larger polycyclic aromatics [4]. All-metal aromatics are a group of metallic rings that also conform to the Hückel (4n + 2) electrons rule. Depending on the type of electrons (s, p or d) delocalized among the metallic core, these all-metal aromatics possess σ, π or δ-aromaticity, respectively. More recently, the voice that the concept of aromaticity should not be extended beyond carbon-based systems is present because they believe that aromatic organometallics are very likely to be fragile. However, the discovery of all-metal aromaticity started from the theoretical predictions of aromaticity for metallocyclopentadienyls [5,6,7,8,9,10]. Later on, several organometallic compounds consisting of metallic aromatic rings have been isolated with fully qualified characterizations [11,12,13]. Before the vivant investigations on triangular all-metal aromatics, A. I. Boldyrev presented general standards to identify the presence of all-metal aromaticity and antiaromaticity [3,14,15]. Meanwhile, Schleyer introduced Nucleus-Independent Chemical Shifts (NICS) in several papers and established a mature system to predict or prove aromaticity by this method [16,17,18]. Ugalde reviewed the earlier developments of all-metal aromatic compounds and encouraged their promising prospects for further utilization [19]. Later on, the advances in all-metal aromaticity were driven forcefully by several groups [20,21,22,23]. However, we are going to pay more attention to triangular all-metal aromatics and specifically summarize these group of organometallics due to its multiple theoretical predictions and existences, rapid developments and exciting application potentials [24,25].

**Scheme 1:**
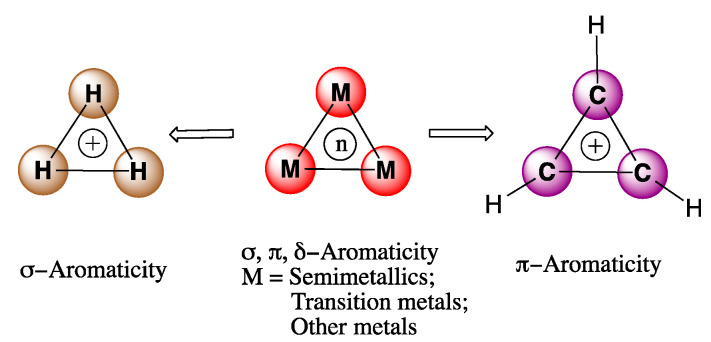
Triangular all-metal aromatic clusters as metallic analogues of σ-aromatic molecule’s [H_3_]^+^ ion or the π-aromatic molecule’s [C_3_H_3_]^+^ ion.

Triangular all-metal aromatics are a group of organometallic complexes that conform to the Hückel (4n + 2) π electrons rule and contain the smallest metallic ring core. In fact, before the successful syntheses and isolation for solid-state triangular all-metal complexes, a great amount of work on theoretical calculations has already predicted the existence of triangular all-metal *σ*, *π* or *δ*-aromaticity among transition metals, semimetallics and other metals, even before 2010 [26,27,28,29,30,31,32,33]. Besides the theoretical predictions, all-metal aromaticity in stable trinuclear clusters has been disclosed by many chemists and these metallic clusters were proved to be either analogues of the *σ*-aromatic molecule’s [H_3_]^+^ ion [34] or analogues of the *π*-aromatic molecule’s [C_3_H_3_]^+^ ion (Scheme 1) [35]. Besides the triangles, the discovery of aromaticity in four-membered all-metal clusters, like [Al_4_]^2−^, also showed considerable research activities [36,37].

Here, in this review, we focus on the theoretical prediction and feasible synthesis of all types of present triangular and sandwich-shaped all-metal aromatics and their rapidly developing applications. The appropriateness of this summarization will boost the developments and applications of this new family of all-metal aromatics and debate Hoffman’s opinion on all-metal aromaticity [38]. We found all-metal aromatics showed a range of potential applications which differ from the public’s misunderstanding that they would be only the laboratory curiosity with gorgeous X-ray structures and limited to further utilizations due to their exaggerated complicated syntheses and low stabilities. As we know, even with the largest ring strain, the simplest aromatic molecular cyclopropenium ion [C_3_H_3_]^+^ is quite stable and most of its metallic analogues showed comparable stabilities. All the known triangular all-metal aromatics will be presented and discussed below, mainly including the syntheses, characterizations and theoretical calculations of isolated triangular all-metal aromatic clusters, predictions of aromaticity in structures theoretically proposed, and their varieties of applications in coordination chemistry, catalytic reactions, and material sciences.

## 2. Experimental Findings of Triangular All-Metal Aromatic Clusters

### 2.1. Triangular π-Aromatic [M_3_]^2−^ (M = B, Al, Ga)

**Scheme 2:**
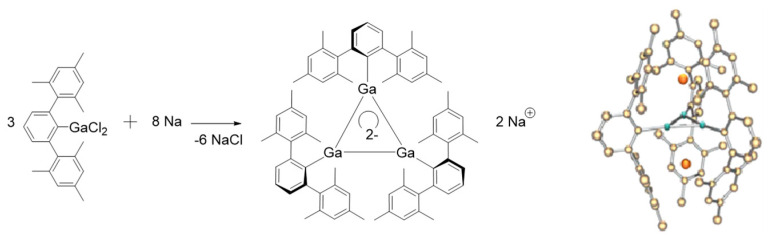
Simple synthesis and X-ray structure of stable [R_3_Ga_3_]^2−^ (R = Mes_2_C_6_H_3_). Hydrogen atoms have been omitted. Adapted with permission from ref. [39] Copyright © 2024, American Chemical Society.

Group 13 triangular all-metal aromatics, which all possess three-centre-two-electron cores, are the earliest investigated triangular systems with aromaticity. In 1995, Robinson and co-workers reported the preparation of cyclogallene, Na_2_[(Mes_2_C_6_H_3_)Ga]_3_ (Mes = 2,4,6-Me_3_C_6_H_2_), which was the first isolated triangular all-metal aromatics. In this large organometallics, three gallium atoms were located on the three termini of the triangular ring core (Scheme 2) [39]. According to the crystal structure solved by X-ray diffraction, Na_2_Ga_3_ exhibited as a peculiar metallic trigonal bipyramid. The metallic core Na_2_Ga_3_ was arranged as two sodium atoms positioned above and beneath the [Ga_3_]^2−^ plane with the bond lengths measured as Ga–Ga: 2.441(1)Å and Ga–Na: 3.220(2) Å, respectively. The authors claimed that the gallium atoms are mainly sp^2^ hybridized according to the electronic properties of cyclogallenes. There was one unoccupied p orbital left for each gallium atom without participating in the formation of a sigma bond. So, each of the two sodium atoms could donate one electron to the unoccupied p orbitals of the gallium atoms to provide the two 2*π*-electrons delocalized on three centres which are required for Hückel’s (4*n* + 2) rule for the determination of aromaticity. Later, more groups found that group 13 M(I) metals could form cationic clusters easily via disproportionation reactions [40].

**Figure 1:**
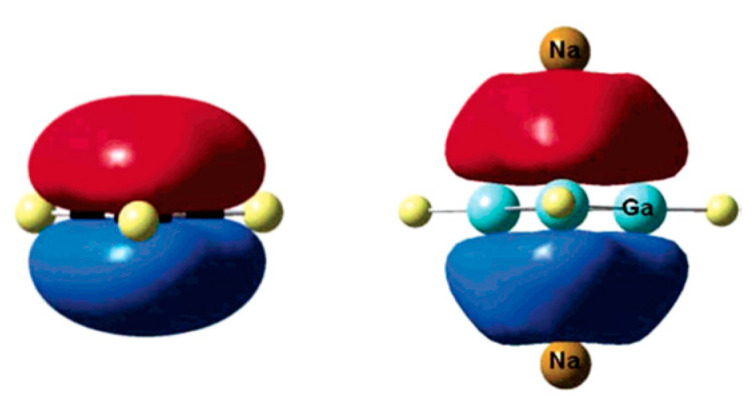
The π-electron clouds (HOMO-1) of the smallest aromatic ring [C_3_H_3_]^+^ and its metallic analogue Na_2_[Ga_3_H_3_]. Adapted with permission from ref. [41] Copyright © 2024, American Chemical Society.

Besides experimental proof, theoretical calculations have been implemented in order to prove the existence of metalloaromaticity in the metallic ring dianion [Ga]_3_^2−^. Through investigation into their electron clouds (Figure 1) [41], the authors certificated that cyclogallenes were metallic analogues of the smallest main group triangular aromatic structure: cyclopropenium cation. The HOMO-1 of the cyclogallene in Na_2_[Ga_3_H_3_] presented extreme similarities to the *π*-electron cloud of the triangular aromatic [C_3_H_3_]^+^ and also possessed the same number of valence electrons. Given the fully recognized “aromatic” property of the cyclopropenium cation, the authors believed that it was justified to claim that triangular “all-metal aromaticity” appeared in these cyclogallene dianions. Combining these experimental findings with their theoretical investigations, Robinson concluded that their isolated Na_2_[Ga_3_H_3_] was the first triangular all-metal π-aromatic structure.

Shortly after the introduction of Na_2_[(Mes_2_C_6_H_3_)Ga]_3_, Robinson and co-workers reported the efficient synthesis and full characterizations of its analogous potassium complex K_2_[(Mes_2_C_6_H_3_)Ga]_3_ (Equation (1)) [42] through the reaction of the corresponding gallium resource with potassium in 2007. K_2_Ga_3_ also contains a similar [Ga_3_]^2−^ aromatic core and bears typical organic ligands. The crystal structure showed that the mean Ga–Ga–Ga bond angle is nearly 60.0° and the Ga–Ga bond distances ranged from 2.4187(5) to 2.4317(5) Å.

3(Mes_2_C_6_H_3_)GaCl_2_ + 8K → K_2_[((Mes_2_C_6_H_3_)Ga)]_3_ + 6KCl (1)

Quantum mechanical self-consistent field and density functional theory (DFT) were authoritative theoretical calculation tools and were exploited to theoretically examine the structural properties of the anionic cores [Ga_3_]^2−^, [GaH]_3_^2−^, Na_2_[GaH]_3_ and K_2_[GaH]_3_. Through full optimizations of their well-defined π molecular orbitals, they found these organometallic species were aromatic, as their calculated Independent Chemical Shifts (NICS) indices were largely negative. With solid experimental and theoretical results, they proved that the triangular metallic cyclogallene dianion, [Ga_3_]^2−^, exhibited definite aromatic behavior [43]. Robinson’s group also proposed that when these three-membered rings were composed of mixed carbon, silicon atoms with gallium atoms could contribute heterocyclic 2π-electron cyclogallene systems which should also be synthetically accessible, stable, and aromatic [44]. Then, based on their huge amount of work in gallium-involved compounds, Robinson summarized short reviews in this field in which quite scientific comments were given on the structures and properties of triangular aromatic organometallic clusters [45].

**Figure 2:**
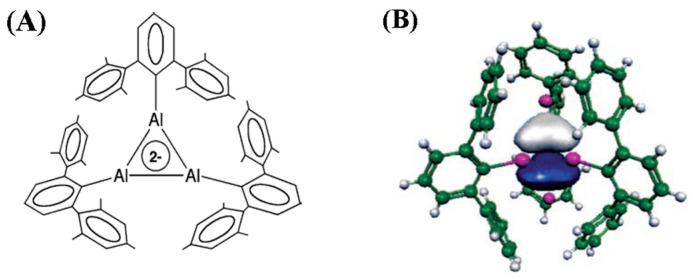
(**A**) The first isolated triangular 2π-electron metalloaromatic aluminium ring Na_2_Al_3_. (**B**) Kohn–Sham orbital representation for the delocalized HOMO-2 of Na_2_Al_3_. Adapted with permission from ref. [46] Copyright © 2024, Wiley-VCH Verlag GmbH & Co. KGaA, Weinheim, Germany.

In 2006, the Na_2_Al_3_ complex, which was an aluminium analogue for the cyclogallenes M_2_Ga_3_ (M = Na, K), was successfully isolated and theoretically demonstrated [46]. Through the rapid reduction process of RAlI_2_ (R = Mes_2_C_6_H_3_) using sodium metal, the three-aluminium core Na_2_Al_3_ was formed in diethyl ether and was successfully isolated after regular work up. According to the Kohn–Sham orbital representation for the delocalized HOMO-2 of Na_2_Al_3_ (Figure 2), the three aluminium atoms in Na_2_Al_3_ could also be considered as sp^2^ hybridized, which was the same mode for the gallium atoms in cyclogallenes. X-ray structure of Na_2_Al_3_ showed the bond length of Al–Al was equal to 2.5202(2) Å on average in the aluminium ring core. Thus, Na_2_Al_3_ was the first characterized aromatic cycloaluminium complex.

**Figure 3:**
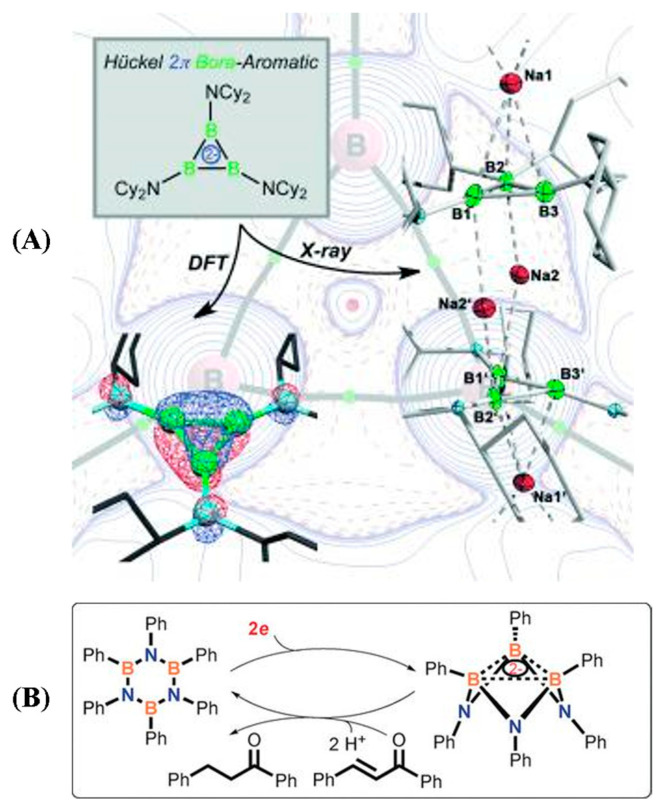
(**A**) The triboracyclopropenyl dianion [B_3_]^2−^: the lightest main-group-element Hückel π aromatic. Adapted with permission from ref. [48] Copyright © 2024, Wiley-VCH Verlag GmbH & Co. KGaA, Weinheim, Germany. (**B**) Aromatic trishomoaromatic (B_3_N_3_Ph_6_)^2−^ structure and its properties in two-electron reduction. Adapted with permission from ref. [48] Copyright © 2024, Wiley-VCH Verlag GmbH & Co. KGaA, Weinheim, Germany.

Besides the heavier elements in group 13, the synthesis for the triboracyclopropenyl dianion was also reported in 2015. The boron-based analogue of the aromatic cyclopropenyl cation belongs to the prototypical Hückel π romatics. [B_3_(NCy_2_)_3_]^2−^ was isolated as its dimeric Na^+^ salt (Na_4_[B_3_(NCy_2_)_3_]_2_ (Figure 3A) [47], which was obtained through reduction of Cl_2_BNCy_2_ by sodium metal. Cyclic voltammetry measurements presented an extremely high oxidation potential (*E*_pc_ = −2.42 V), which was contributed by its good reactivity. The Hückel-type π aromatic character of the [B_3_(NCy_2_)_3_]^2−^ dianion was characterized by single-crystal X-ray diffraction and verified by various theoretical methods. DFT measurements clearly indicated the π aromaticity of the [B_3_]^2−^ core in a similar magnitude to that in cyclopropenium and benzene. In addition, the different ligands stabilized boron-based analogue (B_3_N_3_Ph_6_)^2−^ was synthesized by the simple reduction of B_3_N_3_Ph_6_ using either potassium or rubidium in the 18-crown-6 ether. Similarly, DFT indicated that two electrons delocalized over the three boron atoms in the triangular (B_3_N_3_Ph_6_)^2−^, and the tri-boron core exhibited (π, σ)-mixed homoaromaticity. The reduced (B_3_N_3_Ph_6_)^2−^ could also act as a robust two-electron reductant for unsaturated compounds in reduction reaction (Figure 3B) [48].

**Figure 4:**
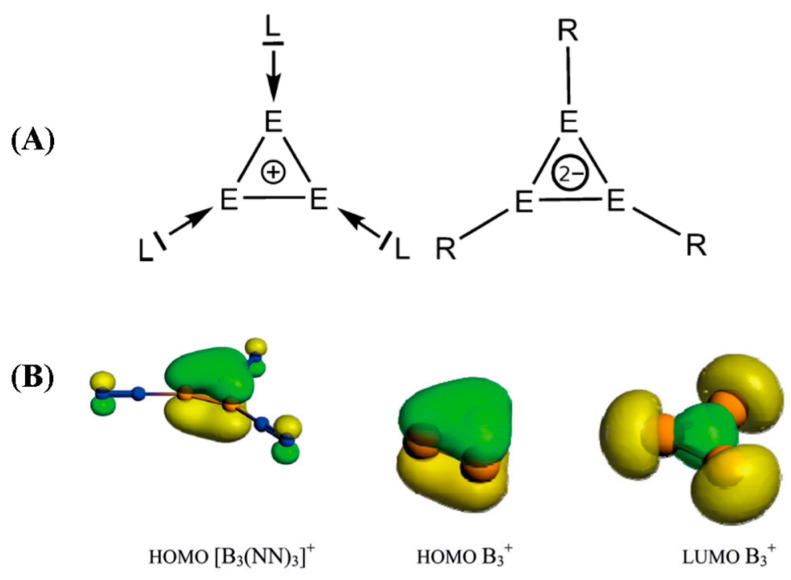
(**A**) Representative two 2π-aromatic systems: cyclic group-13 cation complex [E_3_L_3_]^+^ (E = B) which featuring L→E dative bonds, and cyclic group-13 dianion which feature R-E electron-sharing bonds. (**B**) Plot of molecular orbitals using BP86/TZ2P+: HOMO for [B_3_(NN)^3^]^+^, HOMO for (X^1^A_1′_) B_3_^+^ and LUMO for (X^1^A_1′_) B_3_^+^. Adapted with permission from ref. [49] Copyright © 2024, Wiley-VCH Verlag GmbH & Co. KGaA, Weinheim, Germany.

Besides the π-aromatic core [B_3_]^2−^, Frenking’s group reported another smallest π-aromatic species B_3_^+^ in the [B_3_(NN)_3_]^+^ and [B_3_(CO)_3_]^+^ complexes in 2016. For comparison of the two representative 2π-aromatic systems, cyclic group-13 cation complex [B_3_L_3_]^+^ featuring L→B dative bonds, and cyclic group-13 dianion [B_3_R_3_]^2−^ featuring R-B electron-sharing bonds (Figure 4A) [49]. Spectroscopic identification (mass spectrum and experimental IR spectrum) was obtained for these boron–nitrogen cation complexes. The mass for these cations was formed through pulsed laser vaporization of a boron-11. Fully quantum chemical bonding analysis showed that the ligated adducts were mainly stabilized by the σ-donation of L→[B_3_L_2_]^+^ (Figure 4B). This finding was a new field for ligand-stabilized boron complexes (B_3_L_3_), which could also be extended to main-group adducts with other atoms and ligands in three-membered cycles.

Furthermore, a noble-gas-supported aromatic B_3_^+^ cluster was also obtained by the contribution of strong covalent noble gas–boron bonds [50]. The triangular ion [B_3_]^+^ could also trap rare gases forming circular cationic compounds B_3_Rg_n_^+^ [51]. In fact, besides the [B_3_]^2−^ and [B_3_]^+^ core, fully theoretical evidence confirmed the aromaticity in X_3_^−^ (X = B, Al, Ga) species via Boldyrev’s method [52]. Theoretical studies for the electronic structures, stabilities, and aromaticity in H_2_B_2_XH (X = N, P) molecules and the möbius aromatic planar metallaborocycles were also examined [53,54]. The above all-boron aromatic clusters could play important roles as potential novel ligands or building blocks in chemistry [55].

### 2.2. Triangular π-Aromatic [Si]_3_^+^, [Ge]_3_^+^, [Si_2_C]^+^

**Scheme 3:**
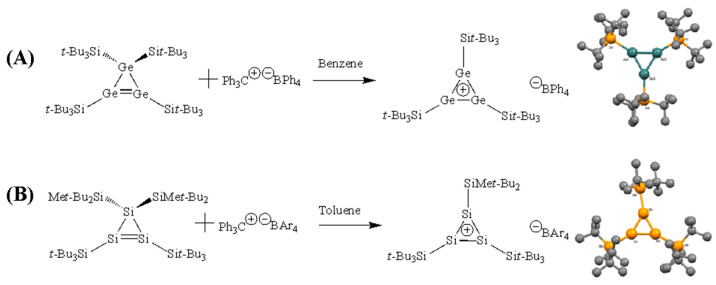
(**A**) Synthesis and crystal structures of aromatic (*t*-Bu_3_SiGe)_3_^+^BPh_4_^−^. (**B**) Synthesis and crystal structures of aromatic [(*t*-Bu_3_Si_2_)_2_(*t*-Bu_2_MeSi_2_)]^+^BAr_4_^−^. Counter anions, crystal solvent molecules and hydrogen atoms have been omitted for clarity (thermal ellipsoids are shown at 30% probability level). Adapted with permission from ref. [62] Copyright © 2024, American Chemical Society.

In addition to group 13 elements, cyclic cations [A_3_H_3_]^+^ (A = C, Si, Ge, Sn, and Pb) were theoretically calculated with curiosities about whether these group of organometallics were going to show any evidence for aromaticity [56,57,58]. The experimental observation of three-membered cations made of the group 14 elements was predicted to be possible [59,60]. Soon, Sekiguchi and co-workers first reported the isolation and characterizations of new tris(tri-*tert*-butylsilyl)cyclotrigermenium tetraphenylborate [*t*-Bu_3_SiGe]_3_^+^BPh_4_^−^, abbreviated as [Ge_3_]^+^ [61]. This [Ge_3_]^+^ system was a free germyl cation with 2π-electrons incorporated in the cyclic core. This [Ge_3_]^+^ complex could be isolated as a type of yellowish solid in the inert atmosphere after the reaction of tetrakis(tri-*tert*-butylsilyl)cyclotrigermene with trityl tetraphenylborate (Scheme 3A). The single-crystal X-ray crystallographic analysis for [Ge_3_]^+^ elucidated that Ge–Ge bond lengths range from 2.321(4) to 2.333(4) Å and the Ge–Ge–Ge bond angles vary from 59.8(1)° to 60.3(1)°. The three Ge atoms were localized in a nearly equilateral triangle. These crystallographic data resemble the cyclopropenium cation. The cyclotrigermenium cation [Ge_3_]^+^ prefers a planar existence due to the electronic properties and steric hindrance caused by the bulky *t*-Bu_3_Si groups. 

In 2005, the same group introduced the first isolation of the persilaaromatic structure [(*t*-Bu_3_Si_2_)_2_(*t*-Bu_2_MeSi_2_)]^+^BAr_4_^−^, abbreviated as [Si_3_]^+^ [62]. Due to the lack of proper precursors, the initial attempts for their syntheses were not successful. With modified methods in the presence of triphenylmethylium tetraarylborate, the desired cyclotrisilene was easily transformed into the targeted cyclotrisilenylium cation (Scheme 3B). According to the crystallographic data, the bond angles in the cyclotrisilenylium cation [Si_3_]^+^ range from 59.76(10)° to 60.20(10)° and the Si–Si bond lengths vary from 2.211(3) to 2.221(3) Å. The internal [Si_3_]^+^ core presented a nearly equilateral triangle. The three substituted silicon atoms are in the same plane within 0.39Å. These experimental characters of [Si_3_]^+^ hinted that it was another triangular all-metal aromatics made of semimetallics.

**Scheme 4:**
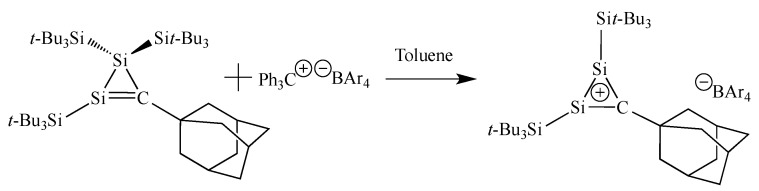
Preparation of substituted [Si_2_C]^+^ by mixing functionalized [Si_2_C] with triphenylmethylium tetraarylborate in organic solvent. Adapted with permission from ref. [63] Copyright © 2024, American Chemical Society.

It is worth mentioning that the synthetic strategies for the robust heteroaromatic cluster [Si_2_C]^+^ were reported by Sekiguchi and co-workers based on the other group’s previous investigations on their analogues [63]. Using (*t-*Bu_3_Si)_2_SiLi_2_ and 1-adamantanecarbonyl chloride in polar organic solvent formed one yellow solid 1,1,2-tris(tri-*tert*-butylsilyl)-3-(1-adamantyl)disilacyclopropene, [Si_2_C], which was examined by X-ray crystallography. The hybridized [Si_2_C] included one Si=C double bond which was equal to 1.745(2) Å and these data resemble the simple cyclopropane. The reaction of [Si_2_C] with triphenylmethylium tetraarylborate gave 1,2-bis(tri-*tert*-butylsilyl)-3-(1-adamantyl) disilacyclopropenylium ion, [Si_2_C]^+^, with great isolated yield (Scheme 4). The tetraarylborate salt of [Si_2_C]^+^ was isolated as yellow crystals and was quite sensitive to air and water. The silicon–carbon hybridized [Si_2_C]^+^ complex represented the first isolable derivative of cyclopropenylium ion, [C_3_]^+^. The mixed heteroaromatic [Si_2_C]^+^ showed an isosceles triangle in crystal structures; similarly, both the cyclopropenyl cation [C_3_]^+^ and its silyl analogue [Si_3_]^+^ were arranged in equilateral triangular cores.

The first three-membered TiSn_2_ ring core was also isolated and fully characterized in 2014 by Kuwabara and coworkers [64]. Normally, the Ti–Sn single bonds in coordinated complexes range from 2.842 to 2.984 Å. However, they found that the Ti–Sn bond lengths in TiSn_2_ ranged from 2.6867(16) to 2.7254(17) Å for Cp_2_Ti[SnC_4_Et_4_]_2_, which was obviously shorter. In addition, the regular bond lengths for Sn=Sn bonds range from 2.575(4) to 2.85126(19) Å; however, the distance between the two Sn atoms in TiSn_2_ was equal to 3.0576(14) Å, which was obviously much longer. All these crystal results implied the possibility of the presence of electron delocalization and aromaticity in the three-membered TiSn_2_ core. Indeed, their following complementary theoretical analysis confirmed that their triangular TiSn_2_ was σ-aromatic. Further, another niobium-necked cluster [As_3_Nb(As_3_Sn_3_)]^3−^ was also experimentally and theoretically examined, and confirmed that the Sn_3_^2−^ core was aromatic [65].

### 2.3. Triangular σ-Aromatic [Au]_3_^+^

**Scheme 5:**
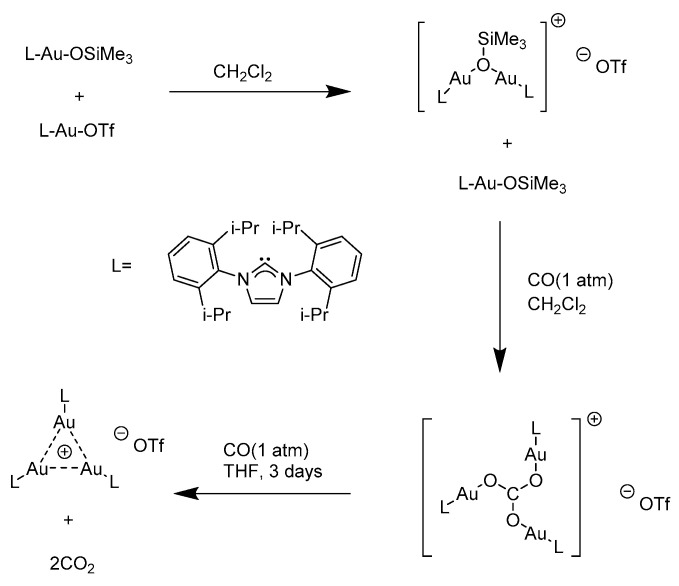
Synthetic route for the cationic trigold(I) cluster and its following reduction to [Au]_3_^+^OTf^−^. Adapted with permission from ref. [66] Copyright © 2024, Wiley-VCH Verlag GmbH & Co. KGaA, Weinheim, Germany.

**Figure 5:**
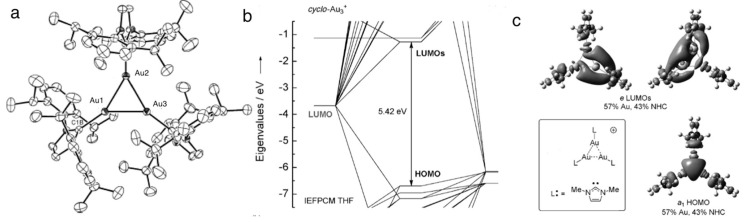
(**a**) Crystal structure of [LAu]_3_^+^ (L = 1,3-bis(2,6-diisopropylphenyl)imidazol-2-ylidene). Selected bond lengths [Å]: Au1–Au2 2.6563 (6), Au1–Au3 2.6633(5), Au2–Au3 2.6438(5); and bond angles [^o^]: Au2-Au1-Au3, 59.603(9), Au2-Au3-Au1, 60.066(15), Au3-Au2-Au1, 60.331(8). (**b**) The partial Kohn–Sham orbital energy diagram of [LAu]_3_^+^. (**c**) Plots of selected orbitals and percentage compositions based on electron densities for [LAu]_3_^+^. Adapted with permission from ref. [67] Copyright © 2024, Wiley-VCH Verlag GmbH & Co. KGaA, Weinheim, Germany.

Using powerful N-heterocyclic carbene (NHC) 1,3-bis(2,6-diisopropylphenyl)imidazol-2-ylidene as the ligand, the first NHC-coordinated triangular tri-gold cluster [LAu]_3_^+^ was synthesized by Sadighi’s group in 2012 [66]. The preparation of [LAu]_3_^+^ was quite simple, with two steps (Scheme 5). First, the monomer [(LAu)_3_CO_3_]^+^OTf^−^ was obtained when [(LAu)_2_OSiMe_3_]^+^OTf^−^ reacted with [LAuOSiMe_3_] under the pure CO_2_ atmosphere. Secondly, in the presence of CO, [(LAu)_3_CO_3_]^+^OTf^−^ underwent reduction and gave the reduced cyclic trimer, [(LAu)_3_]^+^OTf^−^. Crystallographic analysis for triangular [LAu]_3_^+^ cation showed approximate *D*_3_ symmetry with canted imidazolylidene rings (Figure 5a). The [Au]_3_^+^ ring was nearly equilateral, the Au–Au–Au bond angles ranged from 59.603(9)° to 60.331(8)° and the length of the Au–Au bond ranged from 2.6438(5) to 2.6633(5) Å. The three directly connected carbon atoms on the NHC are basically coplanar with the triangular [Au]_3_^+^ core.

Full valence delocalization along the cationic tri-gold core was discovered according to their complementary DFT analysis. The HOMO was composed mainly of the 6s orbitals for gold and the LUMO was composed of a degenerate pair. According to the frontier-orbital energy-level diagram, the calculated HOMO–LUMO gap was quite large (5.42 eV), which hinted at potential inert properties (Figure 5b). Theoretical parameters explained the great stabilities of the complex [LAu]_3_^+^OTf^−^. The plots of the highest occupied (HOMO) and the lowest unoccupied Kohn–Sham orbitals (LUMOs) showed their corresponding electron densities (Figure 5c). Their orbital pictures were analogous to that of aromatic [H_3_]^+^ and their frontier orbitals were echoed by those in [H_3_]^+^. So, this σ-aromatic [Au_3_]^+^ core with only 6s orbitals contributed to the necessary delocalized three-centre-two-electron mode was isolobal to σ-aromatic [H_3_]^+^.

**Scheme 6:**
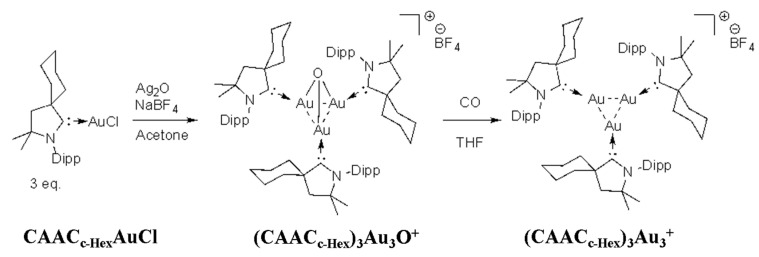
Novel syntheses of trinuclear gold clusters (CAAC_c-Hex_)_3_Au_3_O^+^ and (CAAC_c-Hex_)_3_Au_3_^+^. Adapted with permission from ref. [67] Copyright © 2024, Wiley-VCH Verlag GmbH & Co. KGaA, Weinheim, Germany.

In 2014, Bertrand’s group improved the syntheses of aromatic [LAu]_3_^+^ species with different NHC ligands and attempted applying these peculiar tri-gold clusters in the regular catalytic carbonylation of amines in the presence of CO under mild conditions and presented somehow ordinary reactivities [67]. Based on the known Sadighi’s synthetic strategies, Bertrand introduced another two straightforward synthetic routes for triangular aromatic gold complexes either ligated by three cyclic (alkyl)(amino)carbenes (CAAC) or by mixed ligands (two CAACs plus one phosphine). The first method was treatment of the (CAAC_c-Hex_)AuCl with Ag_2_O in the presence of NaBF_4_ in a polar organic solvent. The intermediate complex (CAAC_c-Hex_)_3_Au_3_O^+^ was obtained as an off-white solid. Then, this oxidized complex was reduced by CO, which yielded pale-yellow solid (CAAC_c-Hex_)_3_Au_3_^+^, abbreviated as [Au]_3_^+^ (Scheme 6). The melting point of [Au]_3_^+^ was 240 °C (dec.), which was an indication of good stability. Indeed, the [Au]_3_^+^ cluster was tested to be robust to oxygen or water, which was an important advantage for further catalytic utilizations.

**Scheme 7:**
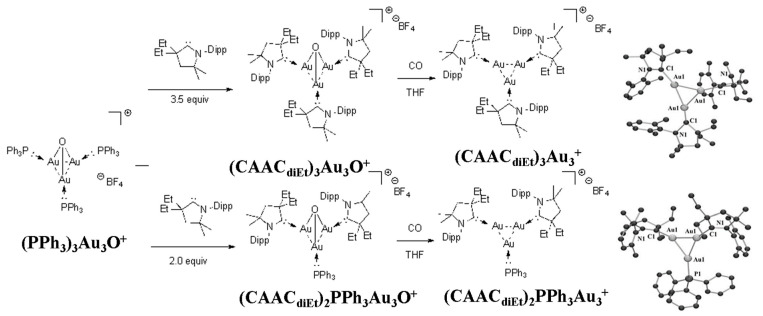
Syntheses and crystal structures of triangular gold clusters (CAAC_diEt_)_3_Au_3_^+^ and the mixed ligands stabilized (CAAC_diEt_)_2_PPh_3_Au_3_^+^. Adapted with permission from ref. [67] Copyright © 2024, Wiley-VCH Verlag GmbH & Co. KGaA, Weinheim, Germany.

The phosphine/NHC ligand exchange possibilities were also investigated using the phosphine-supported μ^3^-oxo cluster (PPh_3_)_3_Au_3_O^+^ as starting material (Scheme 7). Adding excess equivalents of CAAC_diEt_ ligand to (PPh_3_)_3_Au_3_O^+^ gave the μ^3^-oxo complex (CAAC_diEt_)_3_Au_3_O^+^ with good isolated yield. The targeted trinuclear gold ring (CAAC_diEt_)_3_Au_3_^+^ was obtained in nearly quantitative yield after a routine reduction process. When fewer equivalents of CAAC_diEt_ were used, the mixed (CAAC)(phosphine)-ligated complex (CAAC_diEt_)_2_PPh_3_Au_3_^+^ was formed with a moderate yield.

According to the crystallographic analyses, the Au–Au distances in CAAC_diEt_)_3_Au_3_^+^ ranged from 2.6324(8) to 2.6706(8) Å; however, the value was 3.1669(3)–3.3149(3) Å for its oxidized analogue. Comparing the Au–Au bond lengths for these two crystal structures, apparent bond length differences were found. Normally, true covalent bonds are hinted in CAAC_diEt_)_3_Au_3_^+^ due to their unbelievably short metal–metal bond distances. What is worth mentioning is that the CAACs ligand showed stronger σ-donor properties than triphenyl phosphine in the mixed-ligand coordinated structure (CAAC_diEt_)_2_PPh_3_Au_3_^+^. This improved synthetic route provided possibilities for preparation of a variety of polynuclear gold (1/3 valence) clusters through ligand exchanges. Similar to Sadighi’s structure, these mixed ligated clusters [Au]_3_^+^ should also be the analogues of σ-aromatic [H_3_]^+^.

### 2.4. Triangular σ-Aromatic [Zn_3_]^+^, [Zn_2_Cu]

**Figure 6:**
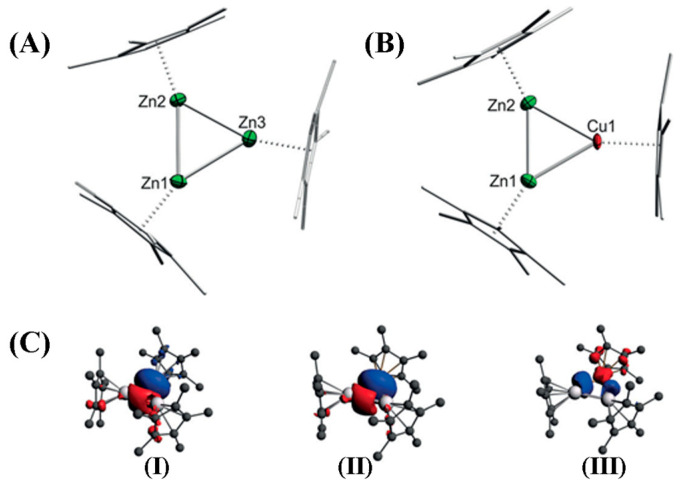
(**A**) Crystal structures of equilateral [Zn_3_]^+^; (**B**) Crystal structures of isosceles [Zn_2_Cu]. (**C**) Deformation densities in triangular [Zn_3_]^+^ and [Zn_2_Cu]. (**I**) [Zn_2_Cp*_2_] [ZnCp*]^+^ σ donation. (**II**) [Zn_2_Cp*_2_] [CuCp*] σ donation. (**III**) [Zn_2_Cp*_2_] [CuCp*] π back-donation. For better understanding, the direction of the charge flow was set from the red part to the blue part. Adapted with permission from ref. [68] Copyright © 2024, Wiley-VCH Verlag GmbH & Co. KGaA, Weinheim.

In 2015, the triangular all-metal aromaticity was extended to Zinc-involved systems by Frenking and Fischer’s group. They synthesized and fully characterized the neutral triangular cluster [Zn_2_CuCp*_3_], abbreviated as [Zn_2_Cu] and the cationic cluster [Zn_3_Cp*_3_][BAr_4_^F^] (Cp* = η-C_5_Me_5_), abbreviated as [Zn_3_]^+^ (Figure 6) [68]. The synthetic procedures were convenient by simply adding [ZnCp*]^+^ and [CuCp*] to [Cp*Zn-ZnCp*] (Carmona’s compound) and stirring. The Zn–Zn bond in [Cp*Zn-ZnCp*] remained after the transformation to the final trimers. The authors claimed that using fluoroaromatic solvents was crucial for their efficient transformation. In addition, Fischer further reported the complementary experimental and theoretical investigations for the pseudo two-electron Cu/Zn clusters [69].

They crystalized both the [Zn_3_]^+^ and [Zn_2_Cu] clusters even sensitive properties were observed for these two ligated metallic trimers. Tri-zinc complex [Zn_3_]^+^ crystallized in the ordinary monoclinic space group *P*2_1_/*c* with two different formula units arranged together (Figure 6A). The neutral compound [Zn_2_Cu] crystallized in the typical triclinic space group *P*_1_^−^ (Figure 6B). The bond angles for [Zn_3_]^+^ were very similar as 59.22(1)°, 59.71(2)° and 61.06(2)º, and for [Zn_2_Cu], the angles were measured as 59.33(2)°, 60.34(3)° and 60.34(3)°. So, the metal atoms in both three-membered core structures presented almost equilateral triangular arrangements. The average distance of Zn–Zn bond in [Zn_3_]^+^ was 2.430(1) Å. Similarly, the two Zn–Cu bonds in isosceles [Zn_2_Cu] were measured to be the same and was equal to 2.381(1) Å. The only Zn1–Zn2 bond in [Zn_2_Cu] was slightly shorter than 2.357(1) Å. All metal–metal bonds in these two complexes are slightly longer compared with their zinc-contained starting material decamethyldizincocene [70].

In order to better understand the bonding interactions of these two triangular clusters, the cationic [Zn_3_]^+^ and its neutral homologue [Zn_2_Cu] were calculated using the *meta*-GGA functional M06L method [71,72]. The nature of the bonding was analyzed by the energy decomposition analysis (EDA) method. They found that the σ donation of [Zn_2_] to [Zn]^+^ into its empty 4s valence orbital was the main constitution for the overall orbital interactions (Figure 6C(I)). In addition, there are two types of leading orbital interactions for the neutral complex [Zn_2_Cu] including the σ-donation from [Zn_2_] to [Cu] and the π back-donation from [Cu] to [Zn_2_] (Figure 6C(II,III)). Both the triangular cores [Zn_3_]^+^ and [Zn_2_Cu] showed valence electrons (cve) delocalization in one cyclic plane and presented apparent implications for σ-aromaticity which was further confirmed by quantum chemical calculations. Similar to the triatomic hydrogen ion [H_3_]^+^, both the [Zn_3_]^+^ and [Zn_2_Cu] clusters revealed a high degree of *σ*-aromaticity.

### 2.5. Triangular Homoleptic [Hg_3_]^4+^

The homoleptic triangular cluster [Hg_3_(μ-dmpm)_4_][O_3_SCF_3_]_4_ [dmpm = bis(dimethylphosphino)methane], abbreviated as [Hg_3_]^4+^ [73], was synthesized and crystalized by Peringer’s group many years after their other brief report on a similar cluster, [Hg_3_(μ-dppm)_3_][O_3_SCF_3_]_4_ [dppm = bis(diphenylphosphino)methane] [74]. Each of the mercury atom in the cluster [Hg_3_]^4+^ represented the 4/3 oxidation state. It was simply synthesized by mixing [Hg_2_]^2+^ with excess dmpm (Equation (2)) or by the reduction of [Hg(Me_2_SO)_6_][O_3_SCF_3_]_2_ in the presence of dmpm and mercury (Equation (3)).

2[Hg_2_][O_3_SCF_3_]_2_ + 4 dmpm →[Hg_3_(μ-dmpm)_4_][O_3_SCF_3_]_4_ + Hg (2)

2[Hg(Me_2_SO)_6_][O_3_SCF_3_]_2_ + 4 dmpm + Hg → [Hg_3_(μ-dmpm)_4_][O_3_SCF_3_]_4_ + 12 Me_2_SO (3)

**Figure 7:**
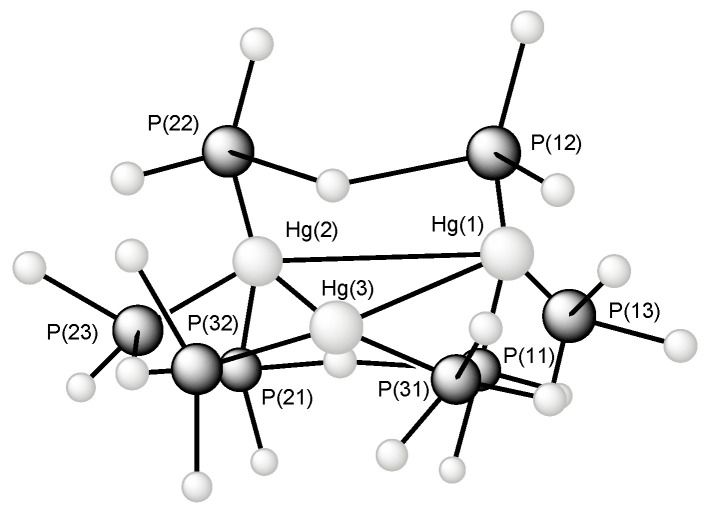
Crystal strucuture of homoleptic triangular [Hg_3_(μ-dmpm)_4_]^4+^. Adapted with permission from ref. [73] Copyright © 2024, Royal Society of Chemistry.

According to the single-crystal X-ray analysis for complex [Hg_3_]^4+^ (Figure 7), this molecule was composed of four CF_3_SO_3_^−^ anions for each [Hg_3_]^4+^ cation, so the oxidation state for each Hg was distributed as +4/3 correspondingly. The cationic [Hg_3_]^4+^ core was presented as a triangular mercury ring with bond lengths measured as Hg–Hg 276.68(14), 295.53(14) and 280.99(14) pm. There are four dmpm ligands involved for three mercury atoms, and the mercury edges are linked to either one or two phosphorous bridges. The three Hg atoms are nearly in a plane. Because of the intramolecular exchange between the two types of dmpm ligands, the complex showed fluxional ^31^P NMR signals at 81 MHz at room temperature. According to the experimental parameters for homoleptic [Hg_3_]^4+^ cation, it was assumed to be another close analogue of the [Au_3_]^+^ and the [Zn_3_]^+^ systems, which are isolobal to σ-aromatic [H_3_]^+^.

### 2.6. Triangular δ-Aromatic [Pd_3_]^+^, [Pt_3_]^+^, [Pd_2_Pt]^+^, [PdPt_2_]^+^

**Scheme 8:**
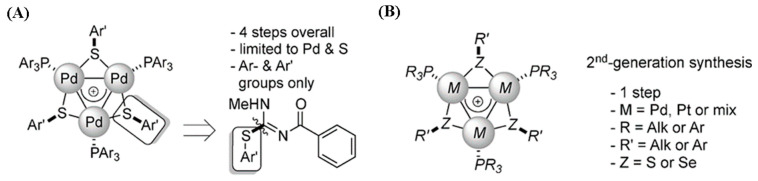
Initial (**A**) and improved (**B**) synthetic routes for aromatic [Pd_3_]^+^, [Pd_3_]^+^ and heteroaromatics [Pd_2_Pt]^+^ and [PdPt_2_]^+^ clusters. Adapted with permission from ref. [76] Copyright © 2024, Wiley-VCH Verlag GmbH & Co. KGaA, Weinheim, Germany.

**Figure 8:**
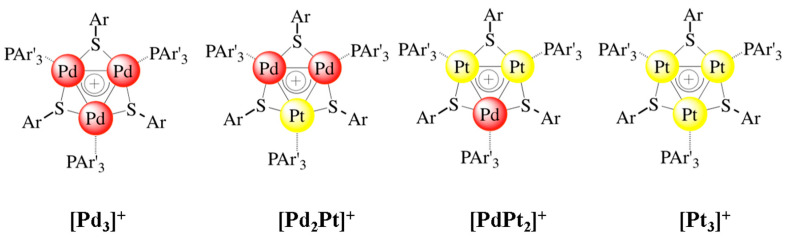
Representative examples for all-metal *δ*-aromatic triangular molecules [Pd_3_]^+^, [Pt_3_]^+^ and heteroaromatics [Pd_2_Pt]^+^, [PdPt_2_]^+^ in which Z = S, anion = SbF_6_, R = 4-ClC_6_H_4_ and R’ = 4-FC_6_H_4_.

In 2014, Malacria’s group reported the mixed-ligand stabilized 44-cve tri-palladium cluster [Pd_3_(μ_2_-SPh)_3_(PPh_3_)]^+^, abbreviated as [Pd_3_]^+^, as the first isolated *d*-block analogue of the π-aromatic cyclopropenyl cation [C_3_H_3_]^+^ [75]. These [Pd_3_]^+^ clusters were firstly obtained by using S-aryl isothioureas as the sulfur source and have further been confirmed to be *δ*-aromatic by theoretical calculations (Scheme 8A). Then, a much-improved method was presented to access the identical tri-palladium cluster [Pd_3_]^+^ and its homoaromatics tri-platinum cluster [Pt_3_]^+^ and mixed-metallic heteroaromatics [Pd_2_Pt]^+^ and [PdPt_2_]^+^ (Scheme 8B) [76]. This new synthetic route was more efficient with almost quantitative conversions without preparing the S-aryl isothioureas. Using this updated method, aliphatic S-bridges and aliphatic phosphorous ligands are compatible and can be installed with high yields. The anionic part for the targeted [Pd_3_]^+^ cluster could be changed simply by hiring different Ag^I^ salts. By replacing disulfide with diselenides, the Se-bridged [Pd_3_]^+^ complex could also be obtained with much lower yields due to its intrinsic lower stabilities. The triangular triplatinum cluster [Pt_3_]^+^ could also be prepared using Pt^0^ precursor Pt(dba)_3_ instead of Pt^II^ species in order to achieve more efficient and more selective transformations [77]. Most importantly, when Pd(0) and Pt(0) precursors were mixed in 2:1 or 1:2 ratios, the peculiar heterobimetallic clusters [Pd_2_Pt]^+^ and [PdPt_2_]^+^ were formed, respectively (Figure 8). The isolated [Pd_2_Pt]^+^ and [PdPt_2_]^+^ clusters were considered the first confirmed triangular all-metal heteroaromatics among all the published Pd/Pt mixed complexes [78]. Both the heteroaromatic structures [Pd_2_Pt]^+^ and [PdPt_2_]^+^ were fully characterized including conclusive High Resolution Mass Spectroscopy (HRMS) and single-crystal X-ray diffraction (XRD).

**Figure 9:**
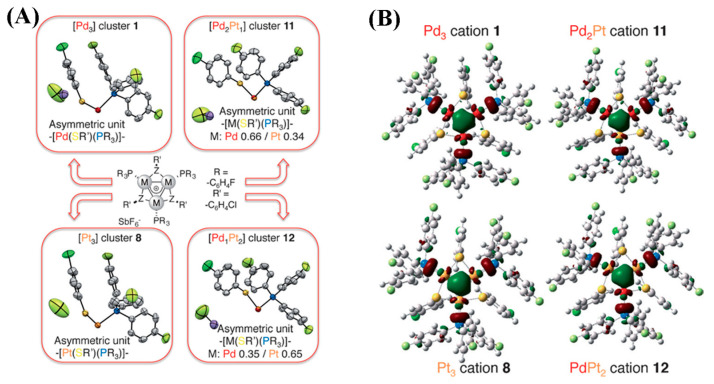
(**A**) Crystallographic comparison for metallic clusters [Pd_3_]^+^, [Pt_3_]^+^ and bimetallic [Pd_2_Pt]^+^ and [PdPt_2_]^+^. (**B**) Delocalized HOMOs for metallic clusters [Pd_3_]^+^ and [Pt_3_]^+^ and bimetallic clusters [Pd_2_Pt]^+^ and [PdPt_2_]^+^. Adapted with permission from ref. [76] Copyright © 2024, Wiley-VCH Verlag GmbH & Co. KGaA, Weinheim, Germany.

For better illustrations and comparisons of the four analogue clusters [Pd_3_]^+^, [Pt_3_]^+^, [Pd_2_Pt]^+^ and [PdPt_2_]^+^ in the X-ray analyses, identical substituent groups were chosen with anion = SbF_6_, R = 4-FC_6_H_4_ and R’ = 4-ClC_6_H_4_, as presented in Figure 9A. After optimization, these four crystalline [Pd_3_]^+^, [Pt_3_]^+^, [Pd_2_Pt]^+^ and [PdPt_2_]^+^ remained triangular, quasi-symmetric structures without any symmetry constraints. Their triangular all-metal core was found to be nearly *D*_3h_-symmetric and their whole structure was almost *C*_3_ symmetry for all these four complexes. The homometallic [Pd_3_]^+^ and [Pt_3_]^+^ triangles were always found to be perfectly equilateral with identical bond angles (60.0°) and exactly the same metal–metal bond lengths no matter the various substituted groups and anions. The Pd–Pd bond distance in [Pd_3_]^+^ core equalled 2.8731(8) Å and the Pt–Pt length in [Pt_3_]^+^ core was 2.8690(4) Å. These data were generally below the sum of Van der Waals radii (3.26 Å).

For the heteroaromatic complexes [Pd_2_Pt]^+^ and [PdPt_2_]^+^, 35% Pd and 65% Pt in [PdPt_2_]^+^ and 66% Pd and 34% Pt in [Pd_2_Pt]^+^ were presented in proportions. The bond angles in these heteroaromatic analogues deviated from 59.5(5) to 60.5(6)°. The averaged bond distance is 2.86(3) Å in [PdPt_2_]^+^ and 2.87(4) Å in [Pd_2_Pt]^+^. These parameters were very close to their homonuclear complexes [Pd_3_]^+^ and [Pt_3_]^+^. Because of the lanthanide contraction, the atomic radii for Pt and Pd are nearly equalized. So, the metal–metal distances are alike for the heterobimetallic clusters [Pd_2_Pt]^+^ and [PdPt_2_]^+^. However, the situation would be much different when two main-group elements were combined.

The most characteristic molecular orbitals for complexes [Pd_3_]^+^, [Pt_3_]^+^, [Pd_2_Pt]^+^ and [PdPt_2_]^+^ were analyzed and compared (Figure 9B). Interestingly, identical bonding HOMOs were found for these four analogues complexes. Not surprisingly, the two bimetallic complexes [Pd_2_Pt]^+^ and [PdPt_2_]^+^ presented reasonable sigmoidal symmetry properties. There is clearly a three-centre-two-electron bond delocalized in their HOMOs. In addition, not only the HOMO but also HOMO-6 and HOMO-36 showed many similarities with the other early transition metal complexes in which *d*-orbital and *δ*-aromatic properties were confirmed through molecular orbitals calculations.

Indeed, the presence of *d*-orbital aromaticity with a typical delocalized three-centre-two-electron bond among the three-membered metallic ring cores of [Pd_3_]^+^, [Pt_3_]^+^, [Pd_2_Pt]^+^ and [PdPt_2_]^+^ was further confirmed by natural bond orbital (NBO) analyses [79] and adaptive natural density partitioning (AdNDP) results [80]. The chemical shifts for these four clusters were always negative (5 Å) in the NICS analyses, which was also a characteristic phenomenon for aromatic structures. The delocalization state of the cyclic electron was also proved by magnetic susceptibilities obtained by SQUID measurements [81,82]. Normally, the χ*T* for a non-aromatic, quasi-symmetric, triangular cluster, [Pd(CO)PPh_3_]_3_ equals 0.04 emuKmol^−^^1^ at 300 K. This value was 0.44 emuKmol^−^^1^ for [Pd_3_]^+^ and 0.48 emuKmol^−^^1^ for [Pt_3_]^+^, respectively, under identical conditions. However, the susceptibilities for bimetallic complexes [Pd_2_Pt]^+^ and [PdPt_2_]^+^ were somehow as low as 0.22 and 0.26 emuKmol^−^^1^, respectively, which was reasonable, because heteroarenes normally show much lower diamagnetic susceptibilities than their analogues homoarenes, even for the molecules made of main group elements [83]. In any case, all four complexes showed much higher values than those of ordinary non-aromatic clusters. The above experimental and theoretical proofs are fully consistent with the presence of *δ*-aromaticity for this group of triangular all-metal aromatics.

### 2.7. Triangular Heteroaromatic [Pd_2_Ru]^+^

**Figure 10:**
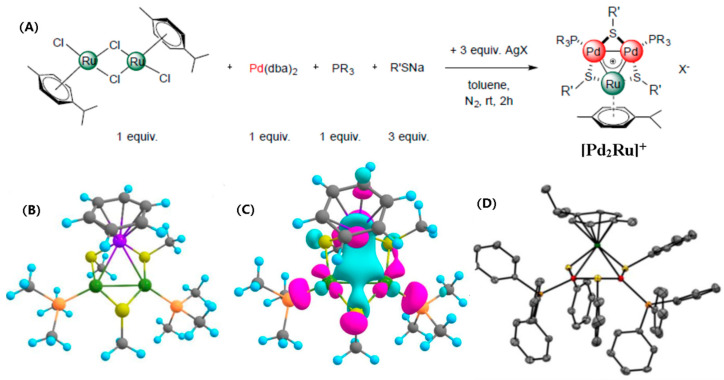
(**A**) Facile synthetic routes for heterobimetallic [Pd_2_Ru]^+^ complexes. (**B**) Simplified model for heteroaromatic [Pd_2_Ru]^+^ complex. Pd-green, Ru-purple, P-orange, S-yellow, C-grey, H-blue. (**C**) The HOMO for the synthesized [Pd_2_Ru]^+^ system. (**D**) Top view for the crystal structure of [Pd_2_Ru]^+^, ellipsoid drawn at 50% probability, Pd (red), Ru (green), P (orange), S (yellow), C (gray). Adapted with permission from ref. [84] Copyright © 2024, Royal Society of Chemistry.

More recently, with the development and increasing interest in the fields of all-metal triangular aromatics, A. I. Boldyrev and coauthors presented another ligand-stabilised heterobimetallic triangular compound [Pd_2_Ru]^+^ (Figure 10) [84]. This complex was air-stable and definitely was the first all-metal aromatic structure composed of noble metals Pd and Ru. Through structural and theoretical analyses, the authors demonstrated both similarities and differences for [Pd_2_Ru]^+^ compared to its parental [Pd_3_]^+^. Indeed, AdNDP and magnetic criteria proved that the [Pd_2_Ru]^+^ core presented obvious overlap between the *d*_*z*^2^_ AO of ruthenium and the *d*_*xy*_ or *d*_*x*^2^_—*y*_2_ AOs of the other two palladium atoms. So, the comprehensive characterisations proved that there is σ-aromaticity among the metal triangular core [Pd_2_Ru]^+^.

However, this structure was actually a byproduct of the reaction to obtain the expected metal triangle [PdRu_2_]^+^ according to the ratio for the two metallic starting reagents. Notably, the two Pd atoms were coordinated with triaryl phosphines, the unique Ru atom was coordinated with *p*-cymene, and the employment of mixed ligands was quite rare compared to the other homo or hetero aromatics. By now, the application for this brilliant complex has not yet implemented, perhaps due to its comparatively low yields. In addition, Boldyrev further summarised the in silico advances in aromaticity and antiaromaticity for bridging transition-metal clusters by bonding and magnetic analyses [10].

### 2.8. Triangular σ-Aromatic [Th_3_]^2−^

**Figure 11:**
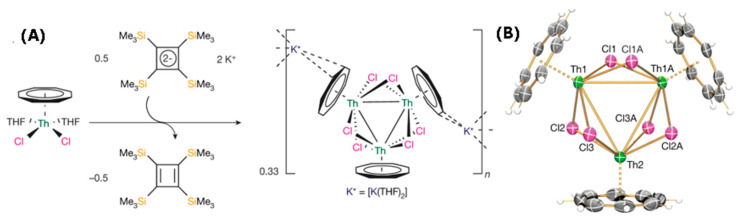
(**A**) Facile synthesis of the tri-Th complex [{Th(C_8_H_8_)(Cl)_2_}_3_]^2−^ by [K_2_{C_4_(SiMe_3_)_4_}] and [Th(*η*^8^-C_8_H_8_)(Cl)_2_(THF)_2_]. (**B**) Crystal structure of [{Th(η^8^-C_8_H_8_)(μ_3_-Cl)_2_}_3_{K(THF)_2_}_2_]_∞_ with 50% probability ellipsoids. Adapted with permission from ref. [85] Copyright © 2024, Springer Nature.

N. Kaltsoyannis and S. T. Liddle synthesized the first σ-aromatic radioactive metal composed tri-thorium cluster {Th_3_(C_8_H_8_)_3_Cl_6_}^2−^, abbreviated as [Th_3_]^2−^ (Figure 11) [85,86]. [Th_3_]^2−^ was obtained through the interaction of [K_2_{C_4_(SiMe_3_)_4_}] with [Th(*η*^8^-C_8_H_8_)(Cl)_2_(THF)_2_]. Single-crystal X-ray diffraction showed that [Th_3_]^2−^ was equilateral, and the average Th-Th bonds distances (3.99 Å) for the triangle was much lower than the sum of Van der Waals radii for thorium (4.74 Å). Indeed, various DFT and NICS analyses showed that the [Th_3_]^2−^ core exhibited a valence-delocalized three-centre-two-electron σ-aromatic character. A short time later, Cuyacot and Foroutan-Nejad made more theoretical comparisons for [Th_3_]^2−^, and questioned the employment of the NICS method and opposed the opinion that the Th–Th bond in the [Th_3_]^2−^ was aromatic. I believe that further studies of this peculiar triangular [Th_3_]^2−^ and the developments for the other predicted triangular metallic aromatics will give a more unified or developed view of triangular all-metal aromaticity.

## 3. Theoretical Predictions of Aromaticity for Triangular All-Metal Clusters

Aside from the above-discussed isolated all-metal aromatics as robust trinuclear crystals or solids, plenty of proposed triangular all-metal structures composed of various transition metals, semimetallics and other metals have been investigated by theoretical calculations in the past decade to predict their potentially possessed all-metal *σ*, *π* or *δ*-aromaticity [87,88,89,90,91,92]. Some typical and representative examples are discussed in the following for their peculiar structures, predicted triangular all-metal aromaticities and potential powerful applications.

### 3.1. d-Orbital Aromaticity in Triangular [M_3_O_9_]^−^, [M_3_O_9_]^2−^(M = W, Mo)

**Figure 12:**
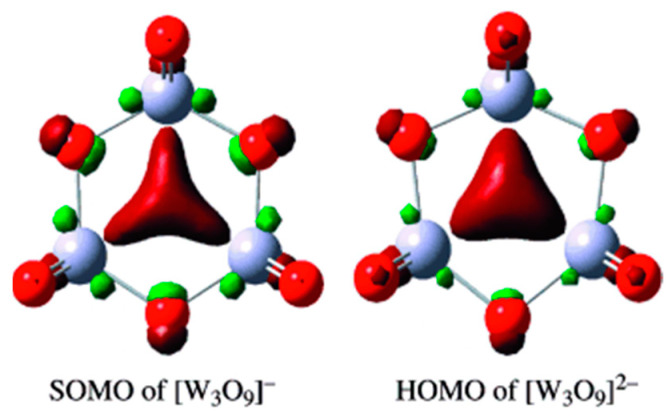
The SOMO for [W_3_O_9_]^−^ and the HOMO for [W_3_O_9_]^2−^. Adapted with permission from ref. [28] Copyright © 2024, Wiley-VCH Verlag GmbH & Co. KGaA, Weinheim, Germany.

In 2005, Wang and co-workers reported the experimental and theoretical proofs of *d*-orbital aromaticity for early transition metal oxide clusters, [M_3_O_9_]^−^ and [M_3_O_9_]^2−^ (M **=** W, Mo) [28]. Both theoretical calculations and photoelectron spectroscopy showed that [W_3_O_9_] and [Mo_3_O_9_] are aromatic anions and presented *D*_3*h*_ symmetric property with a low-lying unoccupied molecular orbital occupied by one or two electrons. The single-occupied molecular orbital (SOMO) for [W_3_O_9_]^−^ and the highest occupied molecular orbital (HOMO) for [W_3_O_9_]^2−^ indicated M–M σ-bonding interactions which were inconsistent with their *d*-orbital aromaticity (Figure 12). The *D_3h_* symmetric [M_3_O_9_]^2−^ dianions were given by adding another electron to this orbital and the three-centre-two-electron (3c–2e) bond was presented.

According to photoelectron spectroscopy (PES) analysis, the calculated resonance energy (about 1.05 eV) for the [M_3_O_9_]^2−^ cluster was comparable to the value estimated for highly aromatic benzene. In addition, the NICS values [93] among the ring centre of [W_3_O_9_]^2−^ and [Mo_3_O_9_] ^2−^ were obtained as −21.5 and −20.5, respectively. So, the [M_3_O_9_]^−^ and [M_3_O_9_]^2−^ clusters described by Wang’s group are peculiar with equal M−M bond lengths, large resonance energies and large negative NICS values, which all suggest that they are highly aromatic species involving fully delocalized metal–metal bonds. This new class of *d*-orbital aromatic species exhibited interesting chemical, electrochemical, and catalytic properties even without isolation and full characterization.

### 3.2. δ-Aromaticity in Triangular [Ta_3_O_3_]^−^

**Figure 13:**
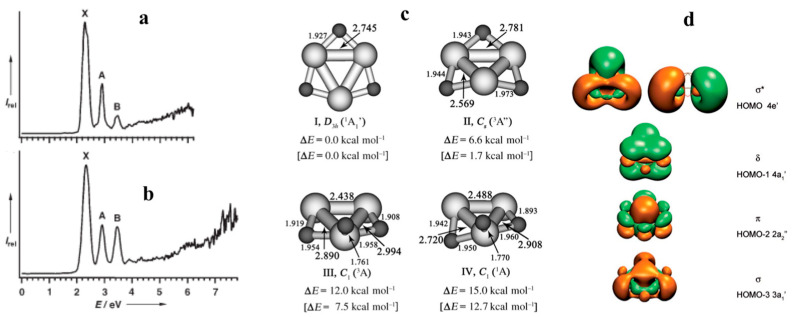
(**a**) Photoelectron spectrum for [Ta_3_O_3_]^−^ under193 nm, A, B, X were representative peaks; (**b**) Photoelectron spectrum for [Ta_3_O_3_]^−^ under 157 nm. (**c**) Optimized and the representative isomers for the [Ta_3_O_3_]^−^. The relative energies Δ*E* and bond lengths for [Ta_3_O_3_]^−^. (**d**) The most representative five valence molecular orbitals in the [Ta_3_O_3_]^−^. Adapted with permission from ref. [98] Copyright © 2024, Wiley-VCH Verlag GmbH & Co. KGaA, Weinheim, Germany.

Many groups have discovered *δ*-aromaticity in metallic systems; the presence of *δ* bonds between identical or different transition-metal atoms indicated that delocalized cyclic *δ* bonds may exist in cyclic transition-metal structures [94]. However, different from valent *s* or *p* orbitals, *d* orbitals are more sterically restricted. As we know, *d*-orbital aromaticity needs substantial *d–d* bonding interactions, so the possibility for the transition metal to participate in forming chemical bonding mainly depends on the type of the transition metals and their spatial coordination circumstances [95,96,97]. In 2007, Wang and Boldyrev confirmed experimental and theoretical evidence for the presence of *δ*-aromaticity in the planar *D_3h_* symmetric triangular cluster [Ta_3_O_3_]^−^ which possessed three delocalized electrons among Ta–Ta bonding [98]. The authors found the presence of not only π-aromaticity but also *δ*-aromaticity contributed by the *d*-bonding interactions in this proposed [Ta_3_O_3_]^−^ cluster according to the photoelectron spectroscopy (PES) [99] analysis under 193 nm (Figure 13a, 6.424 eV) and 157 nm (Figure 13b, 7.866 eV), respectively.

In addition, compared with the common ab initio calculations, both the optimized structures and the most representative electronic transition states for [Ta_3_O_3_]^−^ indicated that this cluster was arranged in a planar *D*_3h_ triangle (Figure 13c). Based on the chemical-bonding analysis, the five most representative valence molecular orbitals participated in the Ta–Ta bonding interactions. Concerning the contributions of the 5d atomic orbitals, the close bonding interactions between the *δ* and *π* orbitals of Ta atoms were apparent (Figure 13d). So, the most representative five valence molecular orbitals demonstrated the delocalization of the *δ* bonds and proved that the [Ta_3_O_3_]^−^ cluster is another example with *δ*-aromaticity. The ligated [Ta_3_O_3_]^−^ cluster has not yet been isolated experimentally.

### 3.3. d-Orbital Aromaticity in Triangular [Tc_3_X_9_]^2−^

**Figure 14:**
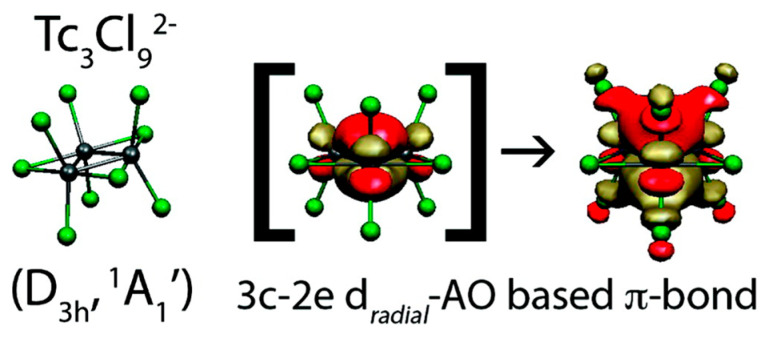
The *d*-orbital-based DFT calculation for trinuclear aromatic [Tc_3_Cl_9_]^2−^. Reprinted with permission from ref. [88] Copyright 2011, American Chemical Society.

Decades ago, scientists started the investigations for trinuclear complexes Re_3_X_9_ (X = Cl, Br, I) and found the electron delocalization character for these potential three-membered metallic cores [30,32,33]. However, the technetium analogues (Figure 14) gained less attention until Weck and coworkers reported their study for the technetium halide complexes [Tc_3_(μ-X)_3_X_6_]^0/1−/2−^ (X = F, Cl, Br, I), which were further confirmed as isomorphous with their rhenium congeners by density functional theory calculations [88]. Even without isolation, the theoretical calculation results via adaptive natural density partitioning demonstrated that the [Tc_3_X_9_]^2−^ complex could exhibit aromaticity contributed by the *d*-orbital-based π electron delocalization over the tri-technetium centre.

### 3.4. Aromaticity/Antiaromaticity of Triangular [Sc_3_]^−^

**Figure 15:**
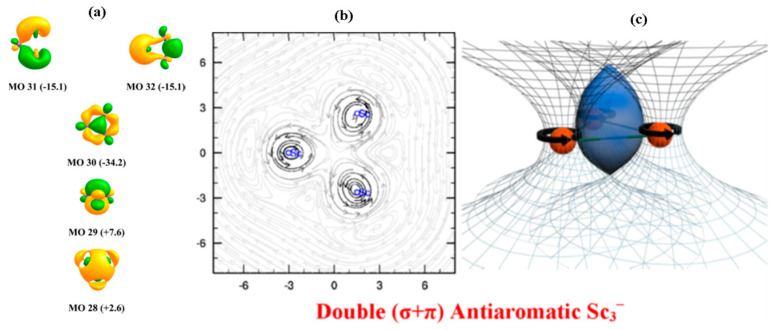
(**a**) Representative molecular orbitals of anionic [Sc_3_]^−^ and their contributions to NICS(0)MO_zz_. (**b**,**c**) The two-zone aromaticity/antiaromaticity character for metallic [Sc_3_]^−^ via the QTAIM-based magnetizabilities. Reprinted with permission from ref. [100] Copyright 2013, American Chemical Society.

Rashidi-Ranjbar, Ruud, Foroutan-Nejad and coworkers reported new insights on the determination of aromaticity in triangular all-metal clusters [Sc_3_]^−^ by giving computational arguments with the help of the ring-current model over local indices [100]. With their efforts, the evidence of the magnetic aromaticity was confirmed by nucleus-independent chemical shifts. Two typical methods for estimating magnetically induced ring currents were employed; the first approach was based on the quantum theory of atoms in molecules (QTAIM) and the second approach was explicit calculation using the magnetically induced current densities. Using the QTAIM-based magnetizabilities principle, they explained the presence of the two-zone aromaticity/antiaromaticity of several 3d all-metallic triangular clusters including Sc_3_^−^, Cu_3_^+^ and Cu_4_^2−^ (Figure 15). According to the classical electromagnetic theory, their theoretical investigations indicated a comprehensive explanation for the anomalous magnetic shielding in different transition metal cluster. The authors also appealed that the nature of magnetic aromaticity/antiaromaticity for all types of triangular transitional all-metal complexes should be determined more comprehensively based on more reliable global indices. 

### 3.5. Aromaticity of Triangular [Al_3_]^−^

**Figure 16:**
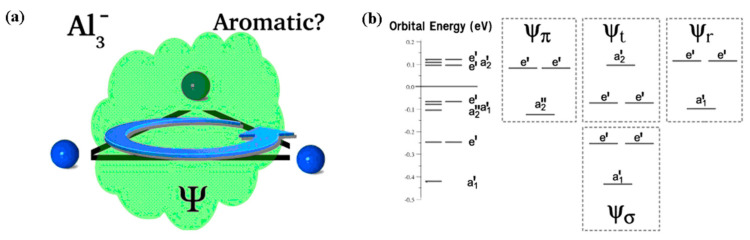
(**a**) The most representative and highly aromatic lowest-lying singlet and triplet states of the all-metal-ring-like cluster Al_3_^−^. (**b**) Valence molecular orbitals of *D_3h_* symmetric cluster Al_3_^−^arranged in increasing energy and grouped into four independent aromatic systems, *Ψ*_σ_, *Ψ_r_*, *Ψ_t_*, and *Ψ*_π_. Adapted with permission from ref. [101] Copyright © 2024, Wiley-VCH Verlag GmbH & Co. KGaA, Weinheim, Germany.

With strong interest, a multiconfigurational high-level electronic structure calculation method was employed to investigate the aromaticity property for the triangular all-metal cluster [Al_3_]^−^ by Ugalde’s group (Figure 16a). Explicit theoretical calculation values indicated that the proposed three-membered-ring-like cluster anion Al_3_^−^ presented the three most representative close low-lying electronic states with clearly different spins, and all these states presented strong multiconfigurational properties. Ugalde and coworkers further studied the aromaticity character of the all-metallic cluster [Al_3_]^−^ using the total electron delocalization method. The related evaluations of the normalized multicentre electron delocalization indices for each state were calculated according to the multiconfigurational wave functions [101]. Combining the multifaceted results, the authors concluded that the lowest-lying singlet and triplet states of Al_3_^−^ were proved to be highly aromatic. In contrast, the closest lowest-lying state (the quintet state) presented much fewer aromatic properties (Figure 16b). Later, theoretical investigations for the d-p hybridized aromaticity, neutral salts and photoelectron spectroscopy of the proposed LaX_2_^−^ (X = Al, Ga, In) clusters were calculated [102]. Near-degenerate molecular-orbital-coupling-induced dual aromaticity in stable open-shell metal clusters was also observed [58]. Furthermore, the possibilities and behaviors of the aluminium trimer during the combination with different superatom clusters were illustrated [103].

### 3.6. Aromatic in Triangular [M_3_]^4+^ (M = Ni, Pd, Pt)

**Figure 17:**
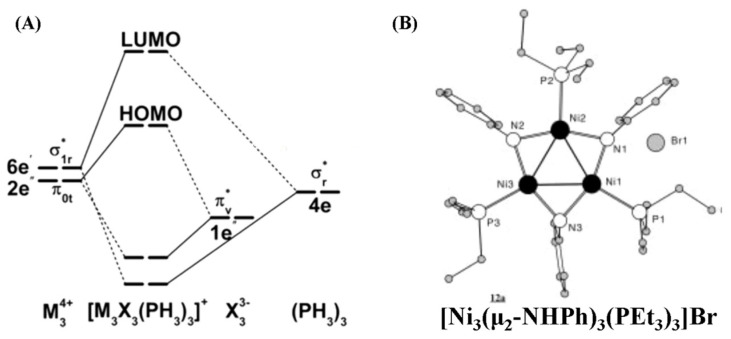
(**A**) The bonding properties of trinuclear clusters [M_3_X_3_]^+^ (M = Ni, Pd, Pt; X = F, Cl, Br, I). Reprinted with permission from ref. [104] Copyright 2017, American Chemical Society. (**B**) Crystal structure for the C_3_ symmetric triangular tri-nickel cluster [Ni_3_(PEt_3_)_3_(μ_2_-NHPh)_2_]Br. Reprinted with permission from ref. [107] Copyright © 2024 WILEY-VCH Verlag GmbH & Co. KGaA, 69451 Weinheim, Germany.

Xiao and Li reported the structural, electronic and bonding properties of a system of triangular trinuclear clusters [M_3_X_3_]^+^ (M = Ni, Pd, Pt; X = F, Cl, Br, I) and explored the electronic and steric effects on the adsorptions of H_2_ and other small molecules (Figure 17A) [104]. The authors presented the chemical bonding models of M_3_ in detail and proposed that the oxidation state of each metal element in the M_3_ cluster was +4/3. In fact, combined with the experimental successes on the isolations of [M_3_]^+^ (M = Pd, Pt) crystals by Malacria’s group, all-metallic σ-aromaticity was also confirmed in these triangular all-metal clusters by theoretical calculations. It was suggested that by changing the energies and compositions of M–M and M–L chemical bonding orbitals, the stability and the catalytic activity of these complexes could be adjusted. The orbital interactions and relativistic effects could be responsible for the active H_2_ dissociative adsorption on these complexes. This finding offered another example of adjusting catalytic properties by tuning different chemical bonding in the all-metal clusters. Actually, similar conclusions have been confirmed experimentally by the highly efficient triangular all-metal aromatic cluster [Pd_3_]^+^-catalyzed internal alkyne reduction reactions. Later, the effects of ligands on bonding stability and σ-aromaticity for Pt nanoclusters were also examined [105]. Modulation of the backside-ligand effect of main-group aromatic ligands (benzene or naphthalene) binding to Pd was also investigated [106].

According to the earlier report of Fenske, treating transition metallic complex NiBr_2_(PEt_3_)_2_ with Li_2_NPh in THF/*n*-Heptane gave nitrogen-bridged triangular tri-nickel cationic cluster [Ni_3_(μ_2_-NHPh)_3_(PEt_3_)_2_]Br, abbreviated as [Ni_3_]^+^ [107]. This [Ni_3_]^+^ cluster was investigated using single-crystal X-ray diffraction (Figure 17B). The crystallographic data proved that [Ni_3_]^+^ was C_3_ symmetric and composed of a Ni_3_-ring with Ni–Ni bond distances ranging from 247.9(1) to 250.6(1) pm, which were the shortest as known and connected with three μ_2_-bridging phenylamido ligands. Besides that, similar to their Pd/Pt analogues, each Ni atom was coordinated with one PEt_3_ ligand. All Ni atoms lay in a plane, resulting in a trigonal-planar structure. Even the authors claimed that this [Ni_3_]^+^ cluster was closer to the non-aromatic palladium compound discussed by Lee and Trogler [108]. However, according to the comprehensive investigations for their Pd/Pt analogues, all geometric parameters for [Ni_3_]^+^ cluster hinted at the possibility of aromaticity in the triangular all-metal core. Taken together with the theoretical calculations, the triangular tri-nickel cationic cluster [Ni_3_]^+^ may be another close analogue to the aromatic systems [Pd_3_]^+^ and [Pt_3_]^+^, which are isolobal to π-aromatic [C_3_H_3_]^+^, whereas further explicit theoretical calculations would be required to give a definitive conclusion on its proposed all-metal π-aromaticity.

### 3.7. Triangular Aromatic Heterobimetallic [Hf_3_]^+^

**Figure 18:**
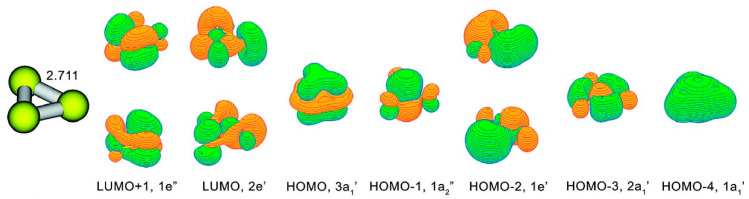
Triple (σ-, π-, and δ-) aromaticity for the Hf_3_ cluster in the Lowest *D_3h_*, ^1^A_1_′ state. Reprinted with permission from ref. [109] Copyright © 2024, American Chemical Society.

Boldyrev presented that the lowest singlet *D*_3*h*_^1^A_1_ structure of [Hf_3_]^+^ is another example of a triple (σ-, π-, and δ-) aromatic system (Figure 18) [109]. An extensive search for the structure of Hf_3_ using the B3LYP/LANL2DZ theory revealed that the lowest triplet and singlet states are *D*_3*h*_^3^A_2_‘ and *D*_3*h*_^1^A_1_‘, respectively. Notably, the triplet state for [Hf_3_]^+^ was the lowest one. However, the authors discovered that these two states are degenerate at the CASSCF/Stuttgart+2f1g level. These results indicated that the singlet state could be the global minimum structure at the higher level of theory. They concluded that the triplet *D*_3*h*_^3^A_2_‘ structure was doubly (σ- and π-) aromatic and the singlet *D*_3*h*_^1^A_1_‘ structure was the first reported triple (σ-, π-, and δ-) aromatic system.

### 3.8. Triangular Aromatic Os_3_N_3_^+/−^

**Figure 19:**
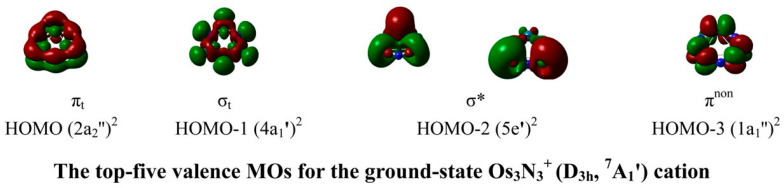
Theoretical calculations for the σ-, π-, and δ- aromaticity in the Os_3_N_3_^+/−^ clusters. Reprinted with permission from ref. [110] Copyright © 2024 Elsevier BV.

Jin’s group investigated the stability, electronic character, and aromaticity of osmium-nitride clusters Os_3_N_3_^+/−^ using DFT (B3LYP methods) (Figure 19) [110]. The theoretical calculations revealed that five types of interactions existed in the hexagonal Os_3_N_3_^+^ (D_3h_, ^7^A_1_′) cation and the regular planar Os_3_N_3_^−^ (D_3h_, ^5^A_1_′) anion. Through detailed molecular orbitals (MOs) analysis, the authors concluded that the cationic Os_3_N_3_^+^ (D_3h_, ^7^A_1_′) and anionic Os_3_N_3_^−^ (D_3h_, ^5^A_1_′) possessed a triple-aromatic character (σ-, π- and δ-aromaticity), simultaneously. It was convinced that the solid structural stability was contributed by the five types of powerful *d*-orbital bonding interactions and triple aromaticity. Based on the aromaticity of the bare osmium trimers, the authors further calculated their binding interactions to group IA/IIA of all-metal series [111].

### 3.9. Aromaticity in Triangular Ir_3_N_3_^+/−^

**Figure 20:**
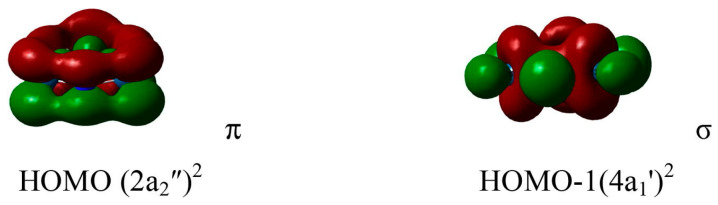
The top-two representative valence MOs for ground state Ir_3_N_3_^2−^ (D_3h_, ^1^A_1_′) motif. Reprinted with permission from ref. [112] Copyright © 2024 Elsevier BV.

In 2017, Ding and coworkers fully investigated the chemical bonding, aromaticity and stabilities of the ground state of iridium low-nitride Ir_3_N_3_^2+/0/2−^ clusters (Figure 20), their metal complexes Ir_3_N_3_M^−/0^ (M = Li, Na, K and Be, Mg, Ca) and bimetallic complexes Ir_3_N_3_M_2_ (M = Li, Na, and K) using DFT calculations [112]. Through detailed molecular orbitals (MOs) analysis, they reveal that the neutral Ir_3_N_3_ (D_3h_, ^3^A_2_’’) cluster possesses σ-, π-, and partial δ- aromaticity, and the Ir_3_N_3_^2−^ (D_3h_, ^1^A_1_’) dianion possesses both σ- and π- aromaticity. They further proved that the pyramidal Ir_3_N_3_M^−/0^ and bipyramidal Ir_3_N_3_M_2_ complexes also possess aromatic characters and preserved the Ir_3_N_3_^2−^ (D _3h_, ^1^A_1_‘) motif.

### 3.10. Aromaticity in Triangular PrB_2_^−^

**Figure 21:**
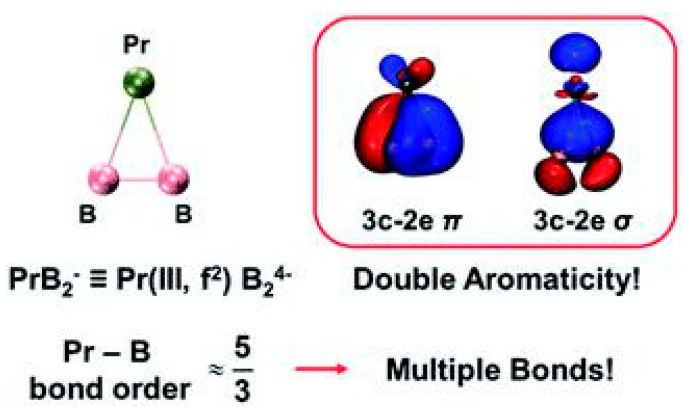
The smallest 4f-metalla-aromatic molecule of cyclo-PrB_2_^−^ with Pr-B multiple bonds. Adapted with permission from ref. [113] Copyright © 2024, Royal Society of Chemistry.

Due to their highly contracted 4f atomic orbitals, lanthanide metals were rarely found in multiple types of aromatic systems. The first example was the lanthanide-boron PrB_2_^−^, which was confirmed as a double-aromatic triatomic molecule based on joint investigations in photoelectron spectroscopy and quantum calculations (Figure 21) [113]. Global minimum structural studies revealed that PrB_2_^−^ possessed a *C*_2v_ triangular structure. Their paramagnetic triplet ^3^B_2_ electronic ground state could be viewed as featuring a B_2_^4−^ and one trivalent Pr(III, f^2^). Chemical bonding analyses concluded that cyclo-PrB_2_^−^ showed multiple Pr–B bonding characters and exhibited σ and π double aromaticity. This smallest 4f-metalla-aromatic system PrB_2_^−^ also highlighted the formation of the rare B_2_^4−^ tetra-anion, completing the isoelectronic C_2_^2−^, N_2_, and O_2_^2+^ series.

According to the Geerlings findings, the metal–metal bonding interactions were much more complicated in the [Li_3_]^+^ system [114]. Using the ring-current map computation, the assumed σ-aromaticity for the triangular [H_3_]^+^ and [Li_3_]^+^ was investigated. The [H_3_]^+^ showed a characteristic diatropic ring current and could be considered as σ-aromatic on the magnetic criterion. However, even with a negative NICS value, [Li_3_]^+^ was concluded to be non-aromatic according to the same criterion because it did not show any global current.

## 4. Catalytic Applications of Triangular All-Metal Aromatic Clusters

For trimetallic systems, scientists are becoming more and more interested in the investigations of their applications and synergistic effects. Clearly, triangular all-metal aromatic clusters contained characteristic delocalized cyclic electrons, metal–metal bonding interactions which determined their good stabilities, and aromaticity, making them become the metallic counterparts of the main-group aromatics. Different from biased opinions on all-metal aromatics for weak stability and rare utilizations, triangular all-metal aromatics were found to show great potential in applications like catalysis, coordination, and material science. In the following part, the most representative examples were summarized.

### 4.1. σ-Aromatic Tri-Gold Cation [Au_3_]^+^ Catalyzed Amine Carbonylation

**Figure 22:**
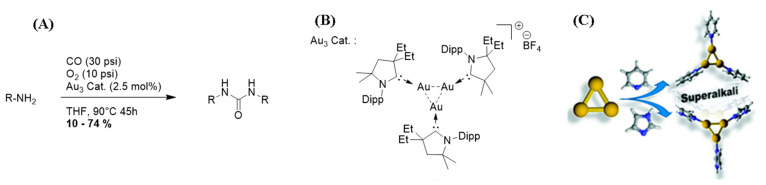
(**A**) Aromatic tri-gold [Au_3_]^+^ catalyzed amine carbonylation in the presence of oxygen. (**B**) Structure of aromatic trigold catalyst [Au_3_]^+^ with substituted NHC as ligand. Adapted with permission from ref. [67] Copyright © 2024, Wiley-VCH Verlag GmbH & Co. KGaA, Weinheim, Germany. (**C**) Proposed superalkali complex made of an all-metal aromatic trigonal Au_3_ core and pyridine (Py) or imidazole (IMD) ligands. Adapted with permission from ref. [126] Copyright © 2024, Royal Society of Chemistry.

The first example referring to the applications of all-metal aromatics in catalytic reactions was reported by Bertrand and coworkers. They presented the syntheses and stabilities for the NHC stabilized aromatic tri-gold complex [Au_3_]^+^ (Figure 22B), and further investigated their catalytic behaviors and plausible mechanisms in the carbonylation reaction [66,67]. Several types of transition metal catalysts presented efficiencies for the reaction of primary amines and carbon monoxide to prepare urea derivatives [115,116,117]. The tri-gold cluster was employed to catalyze this reaction and proved to be a green and selective catalyst (Figure 22A). The triangular all-metal aromatic [Au_3_]^+^ catalyzed carbonylation reaction offered various corresponding substituted urea with mild yields. In order to achieve higher conversions, the catalyst loading reached 2.5 mol% in this reaction. Notably, the isolated yield for the corresponding substituted urea was much lower when the use of the catalyst was decreased. In addition, the substrate scope of this catalytic carbonylation reaction was screened and it was found that fewer spatially hindered amines could be transformed into urea derivatives with higher yields. The authors claimed a peculiar reaction mechanism for this aromatic tri-gold complex [Au_3_]^+^ catalyzed carbonylation of amines. This proposed mechanism involved an oxidation of the valence state of the gold from Au_2_^0^Au^I^ to Au_3_^I^, whereas this proposal demonstrated that these [Au_3_]^+^ clusters lost their aromatic nature as an intermediate. Meanwhile, the more plausible catalytic mechanism is still underway. This reaction also had previously been screened by using the other types of gold-containing catalysts like mono-gold complexes and gold nanoparticles [118,119]. Generally, simple gold complexes presented good activity in this model reaction; for example, Corma et al. showed that small metallic gold clusters could display higher catalytic activities even than small-sized gold nanoparticles and many mono-gold catalysts [120,121,122,123]. Later, the behaviors of σ-aromatic cyclic [M_3_]^+^ (M = Cu, Ag, Au) complexes were investigated when dimethyl imidazol-2-ylidene, isoxazole, pyridine, furan, carbon monoxide and noble gases were introduced for complexation [124]. In addition, the cyclic (R_2_SnAu)_3_ anion was proposed to be stable and showed in-plane σ-Möbius aromaticity [125].

Compared to the usual alkali and alkaline earth metals, analogues super alkalis showed lower ionization energy and possessed peculiar properties. According to the First Principles calculations, predicted superalkali clusters containing triangular all-metal aromatic Au_3_ coordinated with either three imidazole (IMD) or three pyridine (Py) ligands are very possibly to be stable (Figure 22C) [126]. Similar to the properties of superalkali clusters, the calculated ionization energies (IE) for the organometallic complexes, Au_3_(Py)_3_ or Au_3_(IMD)_3_, are quite low. Additionally, the first-order hyperpolarizability calculations proved that some of these coordinated complexes presented non-linear optical properties, which were also similar to the properties of normal super alkali.

**Figure 23:**
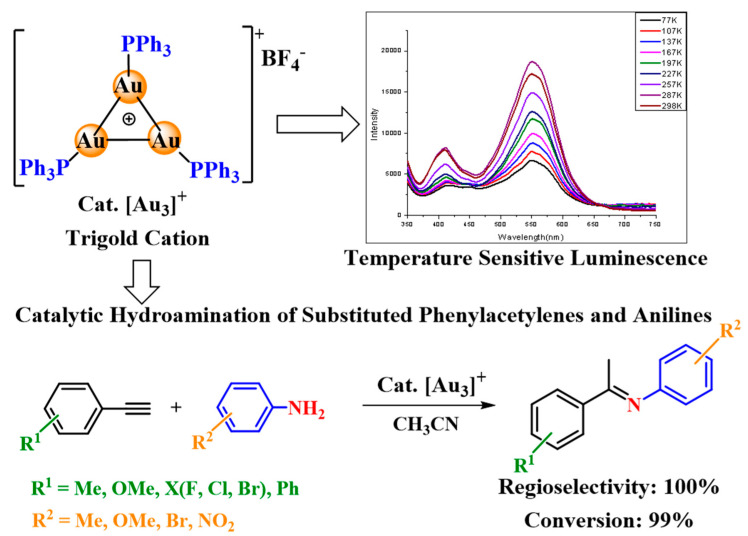
Triphenylphosphines stabilized triangular gold clusters and their application in hydroamination. Reprinted with permission from ref. [127] Copyright © 2024 WILEY-VCH Verlag GmbH & Co. KGaA, 69451 Weinheim, Germany.

Based on the above work on gold clusters, our group synthesized the first triphenylphosphine stabilized triangular tri-gold complex [(PPh_3_Au)_3_]^+^BF_4_^−^ through the facile reduction of μ^3^-oxo complex [μ_3_-O(PPh_3_Au)_3_]^+^BF_4_^−^ (Figure 23) [127]. The cyclic voltammetry of [(PPh_3_Au)_3_]^+^BF_4_^−^ showed one single oxidation process. These tri-gold complexes were further employed in the catalytic hydroamination of phenylacetylenes with anilines. When the loading was decreased to 1.0 mol%, 95% of conversions were obtained. When the catalyst loading further decreased to 0.5 mol%, 83% of isolated yields were achieved in the preparative scale. This application presented great significance in the synthesis of imine-containing pharmaceutical intermediates. Moreover, further investigations of the catalytic mechanisms indicated that these tri-gold complexes played roles as pre-catalysts in the hydroamination.

### 4.2. Aromatic [Pd_3_]^+^ Catalyzed Semi-Reduction of Internal Alkynes

Trinuclear all-metal aromatic [Pd_3_]^+^ should be a good choice for catalytic applications in many types of reactions due to their essential palladium-containing nature and delocalized metal–metal bonding forms. In order to investigate the catalytic properties of all-metal aromaticity in [Pd_3_]^+^, Malacria’s group investigated the catalytic properties of these tripalladium complexes in the classical semihydrogenation reaction of internal alkynes which were used as a model reaction for many types of mono-palladium complexes [128,129,130,131,132]. They found that when palladium clusters [Pd_3_]^+^ were employed to reduce internal alkynes in a hydrogen transfer condition, quantitative (Z)-alkenes were obtained without forming any trace of alkane products [133]. The activity and selectivity for these [Pd_3_]^+^ clusters are comparable with the best mono-palladium peers. At the end of the reaction, the HRMS still detected a trace of the complement catalyst.

**Figure 24:**
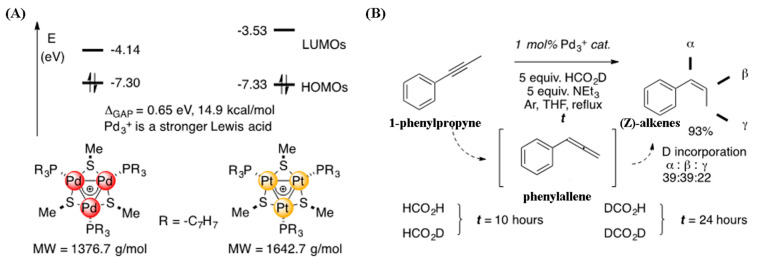
(**A**) Energies of the most representative frontier molecular orbitals of [Pd_3_]^+^ and [Pt_3_]^+^ complexes, values in eV. (**B**) Deuterium-labelling experiment process. Adapted with permission from ref. [133] Copyright © 2024, Wiley-VCH Verlag GmbH & Co. KGaA, Weinheim, Germany.

In contrast to the excellent catalytic behavior of the [Pd_3_]^+^ complex, the aromatic triangular tri-platinum complex [Pt_3_]^+^ showed inert behavior under identical conditions. In order to explain their vastly different catalytic behaviors, the most representative frontier molecular orbitals of the analogues structures [Pd_3_]^+^ and [Pt_3_]^+^ were compared and analyzed (Figure 24A). The LUMO of the active [Pd_3_]^+^ triangle is obviously lower than that of the heavier [Pt_3_]^+^ cluster with the ΔE_gap_ reached to 14.9 kcalmol^−^^1^. On the contrary, according to the literature research on palladium cluster-catalyzed reactions [134,135,136], monoatomic π-acidic Pd-containing clusters are much less electrophilic than their Pt-containing analogues.

The authors also carried out the deuterium-labelling experiment to figure out the possible catalytic pathways (Figure 24B). They found that deuterium atoms incorporated on the terminal carbon of 1-phenylpropyne with [Pd_3_]^+^ complex as the catalyst and the intermediate phenylallene was formed during the catalytic reaction [137]. These findings indicated that the hydrogen transfer step might be rate-determining. In addition, the kinetic studies presented that the hydride-transfer process displayed obvious isotopic effects [138].

**Figure 25:**
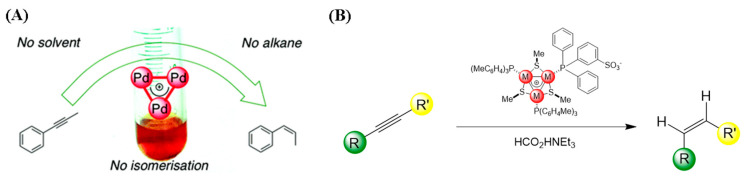
(**A**) Aromatic [Pd_3_]^+^ complexes catalyzed hydrogen transfer reactions of internal alkynes and showed quantitative *cis*-selectivity. Adapted with permission from ref. [139] Copyright © 2024, Royal Society of Chemistry. (**B**) Zwitterionic aromatic [Pd_3_]^+^ complexes catalyzed semi-reduction reaction of internal alkynes. Adapted with permission from ref. [140] Copyright © 2024, American Chemical Society.

Later on, Malacria’s group further discovered that the semi-hydrogenation of internal alkynes could also be catalyzed by the aromatic [Pd_3_]^+^ clusters under neat conditions and gave *cis*-alkenes with quantitative conversions (Figure 25A) [139]. The authors claimed that when 0.03 mol% amount of catalyst was loaded, a large gram scale of the catalytic semi-hydrogenation could be carried out successfully. This group further developed the zwitterionic [Pd_3_]^+^ clusters and employed them in the semi-reduction reaction of internal alkynes as pre-catalysts or as a whole complex. These catalysts propelled the reaction which could be carried out under even neat conditions (Figure 25B) [140]. Generally, triangular all-metal aromatic [Pd_3_]^+^ complexes presented excellent catalytic activities and outstanding selectivity in the semi-reduction of internal alkynes mainly due to their intrinsic aromaticity-induced stability. Aromatic tripalladium complexes could also catalyze inter/intramolecular cascade reactions of 1,6-enynes and carboxylic acids, giving excellent catalytic activities and selectivities [141].

**Figure 26:**
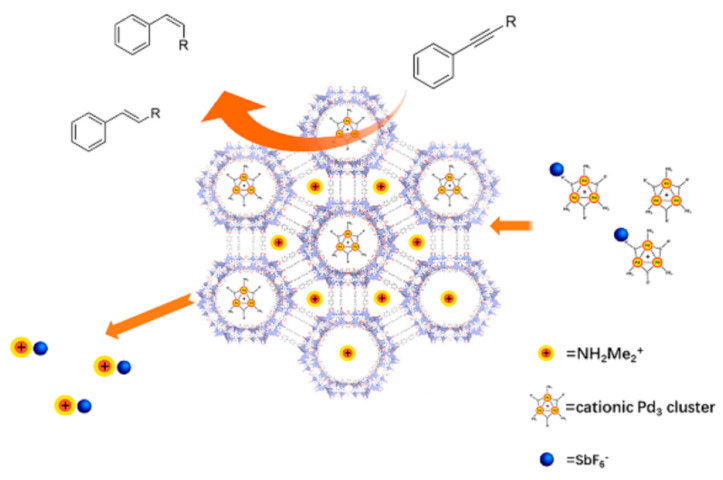
Triangular palladium cations [Pd_3_]^+^ incorporated metal–organic framework and their excellent reactivity in the semi-reduction of internal alkynes. Adapted with permission from ref. [142] Copyright © 2024, American Chemical Society.

Through a regular ion exchange process, anionic MOFs could become frameworks or templates with interesting properties. For example, crystalline MOFs could be further functionalized due to their modular character and novel properties could occur after elaborations. Ma and coworkers presented that by a simple ion exchange process, the trinuclear palladium complex [Pd_3_]^+^ could be introduced to the anionic metal–organic framework (MOFs). In addition, the combined catalytic system was heterogeneous and showed excellent catalytic activity in the classical semi-reduction reaction of internal alkynes (Figure 26) [142]. The authors claimed that the thiol groups from the tri-palladium cluster and the delicate pore sizes of the MOF material significantly minimized the aggregations of palladium clusters during the recyclable catalytic process.

### 4.3. Aromatic [Pd_3_]^+^ Catalyzed Cycloisomerization of 1,6-Enynes and Dienynes

**Figure 27:**
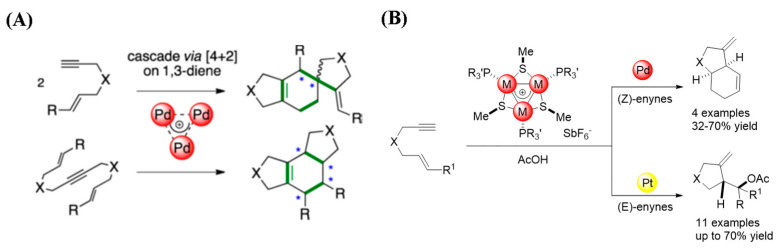
(**A**) Syntheses of functionalized tricyclic cyclohexenes from terminal 1,6-enynes and internal dienynes through aromatic tripalladium complex [Pd_3_]^+^ catalyzed cycloisomerization reaction. Adapted with permission from ref. [143] Copyright © 2024, American Chemical Society. (**B**) Aromatic tripalladium complex [Pd_3_]^+^ catalyzed cycloisomerization of 1,6-enynes in the presence of carboxylic acid. Adapted with permission from ref. [144] Copyright © 2024, Georg Thieme Verlag Stuttgart.

Besides the semi-reduction reaction of internal alkynes, the triangular all-metal aromatic [Pd_3_]^+^ complexes were further applied in the cycloisomerization reactions of substituted internal dienynes and terminal 1,6-enynes and also presented excellent catalytic activity and great selectivity under mild conditions (Figure 27A) [143]. The authors prepared various polycyclic frameworks by modification of substrates and assumed the mechanism of the reaction as a complex cascade process. T Interestingly, the authors later found that the substituted 1,6-enynes showed much different reactive properties when the reaction used carboxylic acid as an additive in the presence of an identical catalyst (Figure 27B) [144]. The catalytic activities of [Pd_3_]^+^ complexes in this cyclisation reaction were comparable to that of much-reported mononuclear Pd(I) and Pd(II) clusters [145,146]. Different from the inertness in the semi-reduction reaction, the analogues tri-platinum catalyst [Pt_3_]^+^ showed good reactivity to cyclize the substrate. However, the authors found that when the analogues tri-palladium complex [Pd_3_]^+^ was used under identical conditions, successive cyclization/double bond shifts happened. So, the tri-platinum catalyst [Pt_3_]^+^ showed a completely different reactive orientation compared with the analogues tri-palladium complex [Pd_3_]^+^ mainly due to their different metallic properties.

### 4.4. Aromatic [Pd_3_]^+^ Catalyzed Coupling Reactions

**Figure 28:**
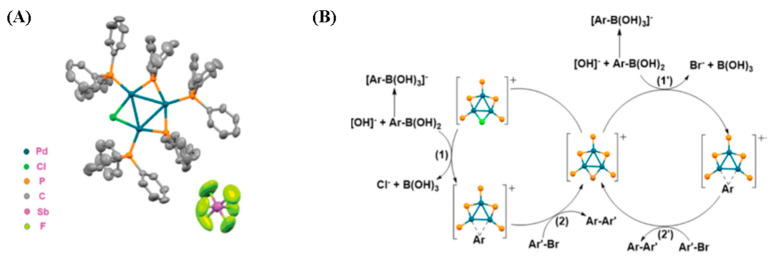
(**A**) The crystal structure of [Pd_3_Cl(PPh_2_)_2_(PPh_3_)_3_]^+^[SbF_6_]^−^. (**B**) Proposed reaction mechanism for the Suzuki reaction catalyzed by [Pd_3_Cl(PPh_2_)_2_(PPh_3_)_3_]^+^[SbF_6_]^−^. Reprinted with permission from ref. [147] Copyright 2017, American Chemical Society.

Besides the above applications in semi-reduction of internal alkynes and cyclisation, [Pd_3_]^+^ also has been successfully applied to various types of carbon–carbon coupling reactions by several groups. Zhu and coworkers reported another type of tri-palladium complex [Pd_3_Cl(PPh_2_)_2_(PPh_3_)_3_]^+^[SbF_6_]^−^, abbreviated as Pd_3_Cl, which was not *C*_3_ symmetry and stabilized by one chlorine atom and two types of aryl phosphine ligands (Figure 28A) [147]. Compared with the other palladium-complex-catalyzed coupling reaction process [148,149], Pd_3_ clusters presented precious C–X (X = halogens) selectivity and excellent efficiency. Pd_3_Cl was proved to be a robust and air-stable cluster during characterization and application. The authors pointed out the σ-aromaticity for the Pd_3_Cl cluster and also claimed that their outstanding stability was mainly contributed by their delocalized 3c–2e Pd–Pd–Pd bonds among the tri-palladium core. One of the most persuasive proofs for the existence of σ-aromaticity was the presence of delocalized 3c–2e σ-bonds.

Later, Pd_3_Cl was further used in homogeneous catalysis with the Suzuki–Miyaura coupling as the model reaction under mild conditions. Among palladium complexes or nanoparticle-catalyzed aryl–aryl bond formation reactions, the Suzuki–Miyaura coupling is one of the most powerful pathways to construct versatile unsymmetrical. The authors investigated the catalytic mechanism of the reaction by monitoring the reaction of simple aryl bromides and arylboronic acids using HRMS and confirmed the presence of possible intermediates in the catalytic circle (Figure 28B). Interestingly, they found the mass for the Ar inserted intermediate through the simple substitution of the Cl atom between two palladium atoms and gave a new understanding of the palladium clusters involved mechanisms of C–C cross-coupling. Additionally, besides aryl bromides, the authors omitted the cases of the aryl chlorides and aryl iodides in this work and did not figure out the halogen selectivity in this case. Even though the mass of the Arinserted intermediate was detected, the proposed catalytic circle was not compliant with the widely recognized palladium-catalyzed coupling reactions.

**Figure 29:**
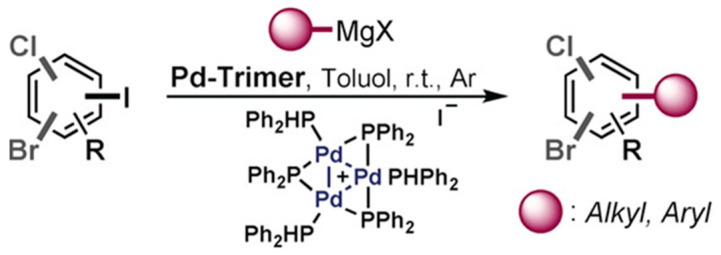
C–I-selective cross-coupling catalyzed by a *C*_3_ symmetric cationic palladium trimer. Adapted with permission from ref. [150] Copyright © 2024, Wiley-VCH Verlag GmbH & Co. KGaA, Weinheim, Germany.

Later, Schoenebeck and coworkers presented another robust palladium trimer using a facile synthetic method [150]. This palladium triangle possessed *C*_3_ symmetric property and was stabilized by only one type of phosphine ligand. Different from the Pd_3_Cl analogue, the Schoenebeck [Pd_3_]^+^ presented unambiguous reactivities and privileged capabilities to C–I bond over C–Br and C–Cl bonds in aryl–aryl bond formations from polyhalogenated arenes and Grignard reagents (Figure 29). The catalytic procedure was classical, using the preformed palladium trimer allowed for quantitative preparation for the arylations and alkylations even for the coupling of steric substrates. Experimental data combined with computational investigations presented the feasibility and capability of the palladium trimer. Even the authors did not mention it, we believe this palladium core [Pd_3_]^+^ presented unique C–I selectivity due to its aromaticity and stability. The cross-coupling catalyst Pd(OAc)_2_/2PPh_3_ could form a dinuclear Pd^I^ complex and further cyclic Pd_3_ clusters during the catalytic process [151].

**Figure 30:**
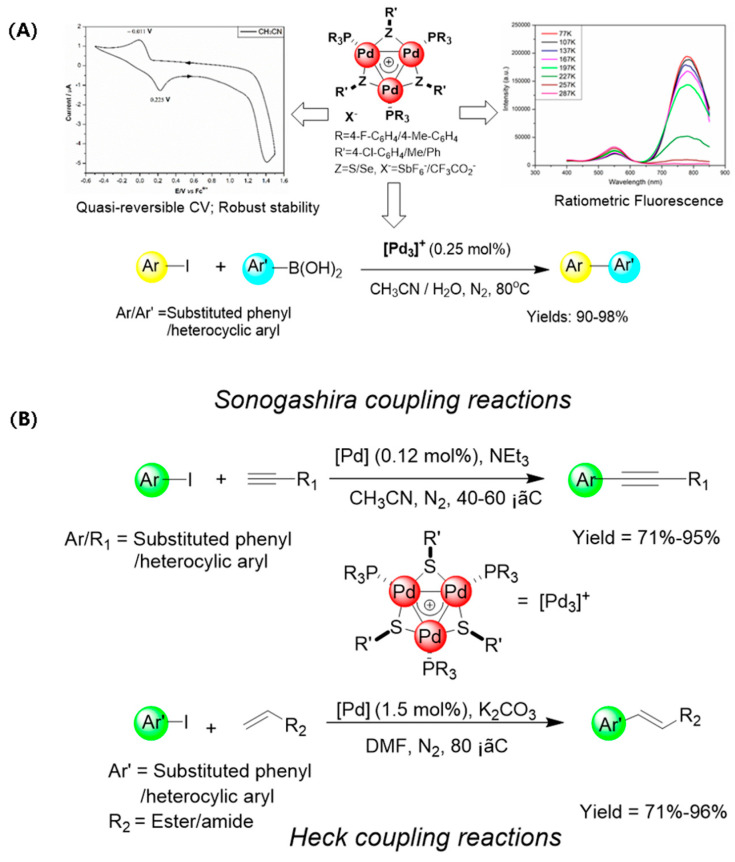
(**A**) Photoelectric properties and catalytic applications for S and aryl phosphine ligated aromatic triangular tri-palladium complexes [Pd_3_]^+^. Adapted with permission from ref. [152] Copyright © 2024, Royal Society of Chemistry. (**B**) Sulfur and aryl phosphine ligated [Pd_3_]^+^ clusters catalyzed Sonogashira and Heck coupling reactions. Adapted with permission from ref. [153] Copyright © 2024, Wiley-VCH Verlag GmbH & Co. KGaA, Weinheim, Germany.

Most importantly, another aromatic tri-palladium complex [Pd_3_]^+^ which was simultaneously stabilized by the sulfur and triaryl phosphine ligands showed outstanding photoelectric properties and was subsequently employed in the Suzuki–Miyaura coupling reaction by our group (Figure 30A) [152]. These *C*_3_-symmetric triangular palladium complexes [Pd_3_]^+^ showed great catalytic activity and exclusive C–X selectivity to aryl iodine thanks to the stability of the catalyst and the mild dissociation energy of the C–I bond. The substrate scope could be extended to thiophene, pyridine, pyrazine, and other common heterocyclic aromatic hydrocarbons. Research on the catalytic mechanism demonstrated that the reaction may involve the intermediate that one sulfur ligand was substituted by the iodine atom monitored by HRMS. A small amount of this tri-palladium complex [Pd_3_]^+^ (0.06 mol%) showed powerful catalytic activity in the gram scale reaction and gave a 93% yield. For the Sonogashira and Heck coupling reactions, this triangular palladium complex [Pd_3_]^+^ also presented unique selectivity for C–I bonds over the other halogen analogues (Figure 30B) [153]. HRMS monitoring for these two coupling reactions showed that the robust [Pd_3_]^+^ remained as a whole at the end of the catalytic process, which proved that the [Pd_3_]^+^ complex was extremely stable.

## 5. Cation–π Interactions of Triangular All-Metal Aromatics

### 5.1. Coordination of Aromatic [Pd_3_]^+^ to Lewis Acids

**Figure 31:**
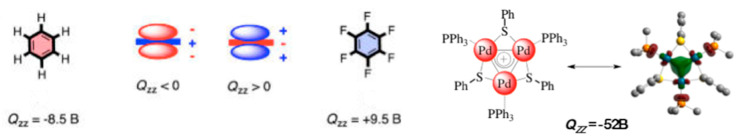
Calculated Quadrupole moments and ion affinities for representative main-group aromatics and aromatic triangular tripalladium cation [Pd_3_]^+^.

Normally, delocalized bonds in main-group aromatics could play the roles as electron donors and form bonding interactions with Lewis-acid species. Indeed, the cation–π interactions are well documented for the main-group aromatics and they play a crucial role in chemistry and biology [154,155,156]. The sulfur atom and aryl phosphine ligand simultaneously stabilized tri-palladium complex [Pd_3_]^+^ has been proven to be aromatic using many types of physical measurements and theoretical calculations. For further extending the applications of [Pd_3_]^+^ in coordination, our group investigated the interactions of the aromatic [Pd_3_]^+^ molecule with a variety of Lewis acids, such as Li(I), Ag(I), Au(I) and Cu(I). With a similar interest, by means of DFT methods, Tsipis predicted the tendency and possibilities of interactions between the ligand-stabilized aromatic triangular gold rings and Lewis acids years earlier [157]. As we know, Lewis acids are possible to form bonding interactions with main group arenes that possess a negative quadrupolar moment perpendicular to their plane (Q_zz_) [158]. For comparison, the calculated Q_zz_ for cationic [Pd_3_]^+^ is −52 Buckingham, and the Q_zz_ of benzene is −8.5 Buckingham, and for hexafluorobenzene is +9.5 Buckingham (Figure 31). By the same calculation model, the calculated Q_zz_ values for the [Pd_3_]^+^ cations were seven times that of benzene. The largely negative values of aromatic [Pd_3_]^+^ suggested that these cationic clusters hold a strong possibility of forming bonding interactions with Lewis acids. However, to the best of our knowledge on heterometallics [159,160,161,162], there were no experimentally isolated complexes to confirm this conjecture.

**Figure 32:**
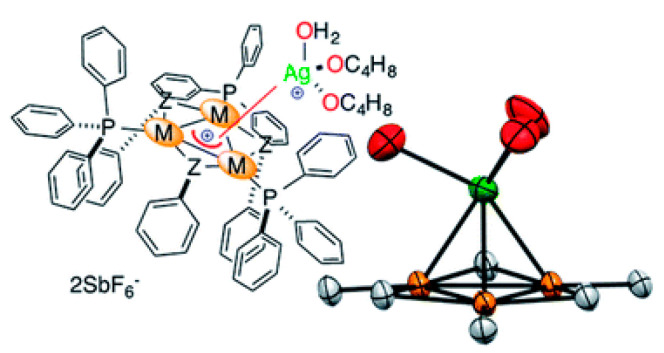
Interactions of all-metal aromatic cationic tri-palladium cores with Lewis acidic cation Ag^+^. Adapted with permission from ref. [163] Copyright © 2024, Royal Society of Chemistry.

In 2017, we reported the first “pyramid” structure of [Pd_3_Ag]^2+^ formed by the coordination of the triangular all-metal aromatic [Pd_3_]^+^ and Lewis acid Ag^+^ (Figure 32) [163]. Cationic [Pd_3_]^+^ can act as donor ligands thanks to their three-centre-two-electron metallic structure with delocalized metal–metal bonds. Driven by the cation–π interaction, the all-metal aromatic ligand [Pd_3_]^+^ and Lewis acid Ag^+^ overcame the unavoidable electrostatic repulsions and formed these “peculiar” cation–cation-coordinated “pyramid” complexes. This result proved that parent all-metal aromatic complexes [Pd_3_]^+^ have the properties of aromatic rings that are crafted with main group elements. Echoed with their main group element counterparts and overcoming the electrostatic repulsions, these noble-metallic rings formed stable bonding interactions with several Lewis acids, such as Ag^+^, Au^+^, Cu^+^, to deliver the corresponding tetranuclear bimetallic complexes with quantitative yields. These novel pyramid tetra-metal complexes [Pd_3_M]^2+^ were fully characterized including HRMS and single-crystal X-ray diffractions. Through comprehensive modelling and experimental techniques for these bimetallic clusters, it was concluded that this bonding mode in [Pd_3_Ag]^2+^ is an original coordination-like one rather than a four-centre-two-electron bond.

### 5.2. Coordination of π-Aromatic B_3_^+^ to Transition Metals

**Figure 33:**
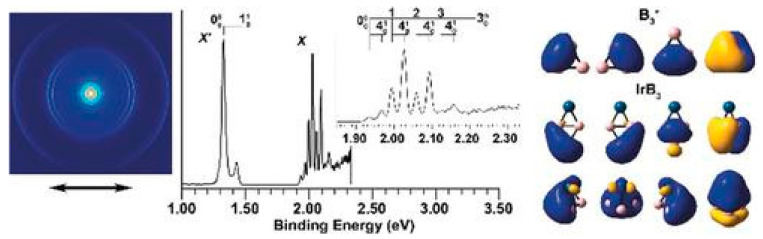
The high-resolution photoelectron image of complexed IrB_3_^−^ structure proved the coordination of π-aromatic B_3_^+^ ring to the transition metal Ir. Adapted with permission from ref. [164] Copyright © 2024, Wiley-VCH Verlag GmbH & Co. KGaA, Weinheim, Germany.

The triangular B_3_ unit is a fundamental bonding template among all boron compounds. Previously isolated B_3_^−^ cluster possessed a *D_3h_* structure and had double (σ and π) aromaticity. Using computational chemistry and high-resolution photoelectron imaging, several chemists investigated the bonding modes between a B_3_ cluster and metallic Lewis acids. For example, Boldyrev and Wang studied the IrB_3_^−^ cluster via high-resolution photoelectron imaging and theoretical calculations. They experimentally observed two isomers with different electron affinities and both structures had a B_3_ ring coordinated with one Ir atom (Figure 33) [164]. The isomer with the higher EA consisted of one B_3_ ring with a bridge-bonded Ir atom (*C_s_*), and the isomer with the lower EA featured a tetrahedral structure (*C*_3*v*_). Chemical bonding analyses showed that the neutral *C*_3*v*_ isomer could be viewed as an Ir–(η^3^-B_3_^+^) complex, in which significant covalent bonding (Ir–B) and weak charge transfer from B_3_ to Ir (ionic bonding) existed. The neutral tetrahedral structure was proved to be very stable. This study provided the experimental evidence and theoretical support for a π-aromatic B_3_^+^ ring coordinating with a Lewis acid or transition metal.

In addition, Wang and colleagues investigated the high-resolution photoelectron imaging of MnB_3_^−^ [165]. Theoretical calculation found that this is a *C_2v_* planar structure in which the Mn is coordinated to only one side of the B_3_ unit. The Mn atom showed weak interactions with the B_3_ unit which still maintained the double aromaticity with small structural changes compared to the bare B_3_ cluster. The present results thus pave the way for the use of suitable triangular all-metal rings as aromatic ligands for a variety of Lewis acids for cation–π interactions. It is anticipated that the introduction of aromatic all-metal cationic metallic rings as a new class of donor ligands is promising for vast innovation in coordination chemistry, materials, and catalysis [166,167,168,169].

## 6. Experimental and Theoretical Developments of Triangular All-Metal Aromatic Sandwiches

**Figure 34:**
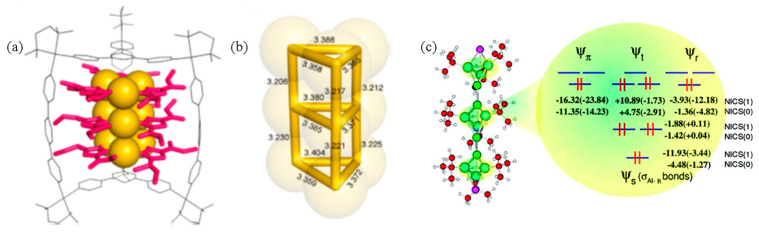
(**a**) X-ray crystal structure of the Au(I) sandwich; (**b**) Highlight of the trigonal prismatic structure for the three-layered Au(I) sandwich. The Au-Au bond lengths are given in Å. Adapted with permission from ref. [171] Copyright © 2024, American Chemical Society. (**c**) Structure and NICS values for sandwich complexes η^3^-Al_3_R_3_. Adapted with permission from ref. [172] Copyright © 2024, American Chemical Society.

A sandwich structure usually means that one metal atom is bounded by two aromatic ligands like ferrocene, which was discovered at the beginning of metallocene chemistry. Now, it has been extended to include aromatic metallic ligands which could also be carbon-free as all-metal-containing layers [170]. In 2010, Fujita and coworkers reported the first three-dimensional all-metal aromatic complex in which metal ions were arrayed in a sandwich shape [171]. The planar polymetallic complexes could be coordinated and assembled as aromatic stacks. For the first time, a crystallographic X-ray of a trigonal prismatic cluster made of three cyclic triangular Au(I) layers was obtained (Figure 34a). By simply mixing the hexa-metallic Au_3_-Au_3_ complex with Ag(I) ion, the Ag-sandwiched complex Au_3_-Ag-Au_3_ could also be obtained. The composition of the initial polynuclear complexes and the shape of the yielded sandwiches determined the exact method of arrangement in the caged clusters (Figure 34b). In fact, based on triangular all-metal mono-layers, the preparation of all-metal aromatic sandwiches was an excellent breakthrough and opened the possibilities for their potential applications in material science as semiconductors.

Besides experimental findings, there are also theoretical proofs for the existence of aromaticity in peculiar all-metal sandwiches. For example, some metallic sandwich complexes made of arraying aromatic triangular layer Al_3_R_3_ (perfluorinated cyclotrialane) were calculated by Mercero and coworkers. For better understanding, their structural and electronic properties were also investigated using density functional theory (Figure 34c) [172]. As we know, the perfluorinated cyclotrialane ring possesses both strong σ and π aromaticities, and it has been confirmed to be a very stable ligand for metal–ligand coordination during the sandwich formation process. In addition, with thorough theoretical calculations, the authors claimed that when the perfluorocyclotrialane ligands were employed to complex the other single metals or metal dimers, the aromaticity of the all-metal triangular ligand remained.

**Figure 35:**
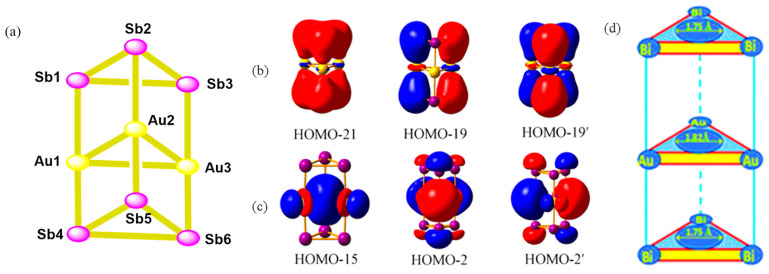
(**a**) Molecular structure for all-metal sandwich complex [Sb_3_Au_3_Sb_3_]^3−^. (**b**) HOMO-21 and HOMO-19/HOMO-19′ showed Sb → Au donation. (**c**) Au 6s-based molecular orbitals: HOMO-15 and HOMO-2/HOMO-2′. Adapted with permission from ref. [173] Copyright © 2024, American Chemical Society. (**d**) Structure of the all-metal aromatic sandwich structure [Bi_3_Au_3_Bi_3_]^3−^. Adapted with permission from ref. [175] Copyright © 2024, Royal Society of Chemistry.

In 2015, Sun, Zhai and coworkers reported the synthesis and isolation of another all-metal aromatic sandwich complex, [Sb_3_Au_3_Sb_3_]^3−^, in which the structure and composition were confirmed by single-crystal X-ray diffraction (Figure 35a) [173]. Quantum chemical calculations demonstrated that there were obvious intramolecular electron transfers among the three all-metal layers. Their valence electrons were clearly rearranged from the cyclo-Sb_3_ layers and Au_3_ interlayers to the Au–Sb bonds. The whole triangular sandwich complex was combined via σ bonding. According to the reversed Hückel rule for aromaticity, the delocalization of the 3c–3e π bonds for each cyclo-Sb_3_ layer proved the presence of aromaticity (Figure 35b,c). Theoretical studies on the electron structures and bonding forms of [Au_3_Sb_6_]^3−^ were also carried out [174]. This new type of all-metal, aromatic sandwich complex was prospected to hold great potential in applications as semiconducting materials.

A short time later, Li and coworkers reported one similar *D*_3h_ symmetric all-metal aromatic sandwich structure, [Bi_3_Au_3_Bi_3_]^3−^; this species was investigated systematically via density functional theory (Figure 35d) [175]. Both molecular orbital analysis and nucleus-independent chemical shift data confirmed that similar to the cyclo-Sb_3_ [Sb_3_Au_3_Sb_3_]^3−^ complex, cyclo-Bi_3_ in [Bi_3_Au_3_Bi_3_]^3−^ possessed both *σ* and *π* aromaticities. However, the cyclo-Au_3_ possessed *σ* and *δ* aromaticity and even weak *π* antiaromaticity. The authors analyzed the bonding nature of each type of bond in this sandwich complex and suggested that the Bi–Bi bond was a nonpolar *σ* covalent bond; the Au–Bi bond is a polar *σ* covalent bond; and the Au–Au bond was attributed to the typical aurophilic interaction. In addition, the intermolecular electron transfer phenomenon between cyclo-Au_3_ and cyclo-Bi_3_ was observed by charge decomposition analysis. Besides that, the robust stability of these complexes could be explained by their large E_gap_ and small Δ e^avg^_R_ which also indicated their potential application as one new type of semiconductor material.

Additionally, the φ-aromaticity for prismatic {Bi_6_}-based clusters was also demonstrated [176]. The aromaticity, electronic structures, and interactions with hydrogens for all-metal aromatic binuclear sandwich complexes were further illustrated [177]. The all-metal binuclear sandwich clusters Al_4_Ti_2_Al_4_ were proved to be high-capacity hydrogen storage structures through multicentre bonds [178]. The four-fold π/σ aromatic sandwich-type Na_6_B_7_^−^ and Na_8_B_7_^+^ complexes featured charge-transfer complexes [179]. Trinuclear mixed-metal sandwich complexes could also construct axially chiral metal skeletons by selective dimerization [180]. The coordination modes and ligand influences for cyclooctatetraene ligated trinuclear Pd3 sandwich complexes were theoretically investigated [181]. Trinuclear Pd metal sheet sandwich complexes have peculiar electronic structures and hold great potential for further functionalization [182].

## 7. Conclusions

Triangular all-metal aromatics are a group of organometallic complexes that possess the smallest ring and conform to the Hückel (4n + 2) rule with all-metal *σ*, *π* or *δ*-aromaticity among transition metals, semimetallics and other metals. These metallic clusters proved to be either analogues of the *σ*-aromatic molecules [H_3_]^+^ ion or analogues of the *π*-aromatic molecule’s [C_3_H_3_]^+^ ion. As summarized in the review, the syntheses, characterizations and theoretical calculations for isolated robust triangular all-metal aromatic clusters like [Ga_3_]^2−^, [Al_3_]^2−^, [B_3_]^2−^ [Si]_3_^+^, [Ge]_3_^+^, [Au]_3_^+^, [Zn_3_]^+^, [Pd_3_]^+^, [Pt_3_]^+^, [Th_3_]^+^, [Pd_2_Ru]^+^, [Pd_2_Pt]^+^ and [PdPt_2_]^+^ have been demonstrated. Theoretical calculations have predicted the existence of multiple types of aromaticity in proposed structures like [M_3_O_9_]^−^ and [M_3_O_9_]^2−^ (M **=** W, Mo), [Ta_3_O_3_]^−^, [Tc_3_X_9_]^2−^, [Sc_3_]^−^, [Al_3_]^−^, [M_3_]^4+^ (M = Ni, Pd, Pt), [Hf_3_]^+^, Os_3_N_3_^+/−^, Ir_3_N_3_^+/−^, and PrB_2_^−^. Experimental and theoretical developments of all-metal aromatics involved in sandwiches are well developed. The aromaticity of the all-metal triangular ligand in the sandwiches could also remain. The isolation of complex aromatic sandwiches will pave the way for the synthesis of entirely new classes of complexes and hold potential technological applications as semiconductors.

The first σ-aromatic cation [Au_3_]^+^ showed clear activity in the amine carbonylation reaction and provided modest yields. Comparatively, trinuclear all-metal aromatic [Pd_3_]^+^ proved to be a strong candidate for catalytic applications in several optimized catalytic systems like semi-reduction of internal alkynes to deliver *cis*-alkenes with almost complete selectivity on a gram scale with very low catalyst loadings. Powerful activity was also obtained in the cycloisomerization of terminal 1,6-enynes and internal dienynes under mild conditions and Suzuki–Miyaura, Sonogashira, and Heck C–C cross-coupling of aryl halides and arylboronic acids under mild conditions. By now, the all-metal aromatic cationic metal rings were applied in coordination chemistry successfully and used as a new class of donor ligands for a variety of Lewis acids through cation–π interactions. This summarised review will propagate the importance and developments of triangular and sandwich-typed all-metal aromatics and the prospect that this group of peculiar structures could participate in more types of in-depth applications in coordination chemistry, catalysis, and material science.

Above all, many types of triangular and sandwich-typed all-metal aromatics were synthetically isolated or theoretically predicted, and the types of metals in three-membered circles possessing aromaticity were still very limited compared to the integral metal species on the periodical table. By now, besides the three-membered circles, larger aromatic circles (four-, five-, six-membered) composed of pure metals were rarely discovered. In fact, most of the successful syntheses originated from unexpected discoveries rather than purposeful preparations and most of the designed syntheses routes cannot be achieved due to their exclusive metallic properties and subtle coordination relationships with ligands. Besides the enthusiasm for their peculiar structures of triangular and sandwich-typed all-metal aromatics, their applications in coordination chemistry, catalysis, and material science could be developed and carried forward in the future decades.

## Data Availability

No new data were created.
